# Effect of Crohn's disease mesenteric mesenchymal stem cells and their extracellular vesicles on T‐cell immunosuppressive capacity

**DOI:** 10.1111/jcmm.17483

**Published:** 2022-09-01

**Authors:** Neda Dadgar, Jessica Altemus, Yan Li, Amy L. Lightner

**Affiliations:** ^1^ Department of Colorectal Surgery Digestive Disease Surgical Institute Cleveland Ohio USA; ^2^ Department of Inflammation and Immunity Lerner Research Institute Cleveland Ohio USA

**Keywords:** creeping fat, Crohn's disease, cytokines, mesenchymal stem cells, murine colitis model, T cells

## Abstract

Crohn's disease (CD) is a chronic inflammatory disease of the gastrointestinal intestinal tract and has characteristic hypertrophic adipose changes observed in the mesentery. To better understand the role of the mesentery in the pathophysiology of Crohn's disease (CD), we evaluated the immunomodulatory potential of mesenchymal stem cells (MSCs) and their secreted extracellular vesicles (EVs) derived from Crohn's patients. MSCs and EVs were isolated from the mesentery and subcutaneous tissues of CD patients and healthy individuals subcutaneous tissues, and were analysed for differentiation, cytokine expression, self‐renewal and proliferation. The varying capacity of these tissue‐derived MSCs and EVs to attenuate T‐cell activation was measured in in vitro and an in vivo murine model. RNA sequencing of inflamed Crohn's disease mesentery tissue revealed an enrichment of T‐cell activation compared to non‐inflamed subcutaneous tissue. MSCs and MSC‐derived EVs isolated from Crohn's mesentery lose their ability to attenuate DSS‐induced colitis compared to subcutaneous tissue‐derived cell or EV therapy. We found that treatment with subcutaneous isolated MSCs and their EV product compared to Crohn's mesentery MSCs or EVs, the inhibition of T‐cell proliferation and IFN‐γ, IL‐17a production increased, suggesting a non‐inflamed microenvironment allows for T‐cell inhibition by MSCs/EVs. Our results demonstrate that Crohn's patient‐derived diseased mesentery tissue MSCs lose their immunosuppressive capacity in the treatment of colitis by distinct regulation of pathogenic T‐cell responses and/or T‐cell infiltration into the colon.

## 1 INTRODUCTION

Crohn's disease (CD) is a chronic idiopathic gastrointestinal disorder characterized by transmural intestinal inflammation resulting in inflammatory, penetrating and stricturing phenotypes. Pathognomonic for CD is the hyperplastic mesenteric adipose tissue found wrapping around the bowel wall predominately at sites of intestinal damage in CD, otherwise called creeping fat.^1^ There has been a recent growing interest in the role of the mesentery in the pathophysiology of CD; rather than disease starting at the mucosa of the small bowel, perhaps the mesentery is driving inflammation rather than being an innocent bystander.^2^ In fact, when an extended mesenteric resection is performed at the time of ileocecal resection for CD, disease recurrence is significantly reduced, and this reduction in recurrence is significantly associated with the degree of fat wrapping identified at the time of surgical resection.^3^

Mesenchymal stem cells (MSCs) and extracellular vesicles (EVs) Derived from MSCs, multipotent non‐haematopoietic progenitor cells, isolated from adipose tissue are thought to be an immunomodulatory and anti‐inflammatory cell via paracrine mediated secretion of growth factors, cytokines, and antifibrotic or angiogenic mediators.^4^, ^5^, ^6^, ^7^, ^8^, ^9^, ^10^, ^11^, ^12^, ^13^ Specific to CD is the emerging data of MSCs as EVs an advanced therapeutic for perianal fistulizing CD, a phenotype of CD that is notoriously difficult to treat.^14^, ^15^ Several phase I, phase II and phase III clinical trials including over 300 patients have now demonstrated safety and improved efficacy of MSCs for perianal CD as compared with conventional biologic and surgical therapy. While the reason for this efficacy has not been fully elucidated, the immunomodulatory properties of healthy MSCs directly affect immunologic pathways of importance to CD, including the increase in T regulatory cells and suppression of pro‐inflammatory cytokines.^16^, ^17^ Lesion tissue of Crohn's disease patients is enriched with CD^4+^ T cells, which cause blocking and cell‐depleting of antibodies, and it causes higher number of Th17 cells with a pro‐inflammatory/cytotoxic phenotype, the importance of Th17 subset can be addressed according to effect of MSCs derived from patients with CD.^18^, ^19^ Effect of MSC‐derived EVs on IBDs was more focus on the roles of extracellular vesicles in health and disease, with a focus on immune regulation and intestinal barrier integrity and showed the anti‐inflammatory effect of them on IBDs.^20^, ^21^, ^22^

It is critical to note that MSCs' original tissue source and anatomical residence affects their immunosuppressive function.^23^, ^24^, ^25^ Given the characteristic adipose changes seen in Crohn's mesentery, we sought to evaluate the differences in MSCs derived from Crohn's mesentery versus subcutaneous tissue to determine whether mesenteric adipose‐derived MSCs may play an active role in the pathophysiology of CD. Given that MSCs have shown a strong immunosuppressive function in T‐ and B‐cell‐derived conditions^26^, ^27^, ^28^, ^29^ and that most of the pathophysiological changes in CD inflamed tissue can be related to the effects of Th1 and Th17 cells,^30^, ^31^, ^32^, ^33^, ^34^, ^35^, ^36^, ^37^ we sought to focus our investigation on MSC‐specific changes in T‐helper (Th) cells in an in vitro and in vivo model of CD. We hypothesized that MSCs derived from hyperplastic mesentery adipose tissue surrounding diseased Crohn's intestine would have a more pro‐inflammatory phenotype suggesting a role for MSCs in ongoing disease progression in CD.

## 2 MATERIALS AND METHODS

### 2.1 Institutional Review Board

The use of human tissue was approved by and performed in accordance with the Cleveland Clinic Institutional Review Board (IRB) (IRB# 19–908). The colorectal surgery biobank is a patient‐identified repository for IBD, colorectal cancer and control (i.e. hernia, diverticulitis, functional bowel disorders) patients. At the time of operative intervention, surgical specimen tissue, subcutaneous adipose tissue, blood and stool may be collected. Adult patients with preoperative consent are included.

### 2.2 Human MSC Isolation

Following patient consent at preoperative surgical visits to participate in the colorectal surgery biobank, healthy individual as control (from hernia patients without inflammation) and CD adult surgical patients' subcutaneous adipose tissue and mesenteric adipose tissue were obtained at the time of surgical resection. Detailed patient information is shown in Table 1. Adipose tissue specimens were transferred in sterile containers directly from the operating room to the biosafety cabinet within 20 min of resection. The specimens were rinsed in sterile PBS until all surface blood was cleared. Adipose‐derived MSCs were isolated from both subcutaneous adipose tissue (control, CD) and mesenteric adipose tissue (CD) following the protocol described in previous studies with minor modifications.^26^, ^27^, ^28^ Briefly, the adipose pearls were micro‐dissected, washed in phosphate buffer saline (PBS), minced and digested with 0.25% type I collagenase (Gibco Life Technologies) at 37°C for 60 min under constant shaking. After removal of the supernatant, cells were suspended and cultured in xeno‐free, serum‐free MSC NutriStem® XF Medium (05–200‐1A‐KT, Biological Industries USA) at 37°C/5% CO_2_. The cultured MSCs were grown to 85–90% confluency in T75 tissue culture flasks before passaging. MSCs were finally harvested at passage 3 to 4. For mouse intraperitoneal injection, MSC pellets were resuspended in PBS.

**TABLE 1:** Patient characteristics

Patient	Diagnosis	Age	Sex	BMI	Phenotype of CD	Corticosteroids at surgery	Monoclonal antibody at surgery
Patient 1	Crohn's disease	62	F	25.3	Stricturing	Yes	Yes, Certolizumab
Patient 2	Crohn's disease	50	F	50.8	Inflammatory	No	No
Patient 3	Crohn's disease	29	F	24.6	Inflammatory	No	Yes, Ustekinumab
Patient 4	Crohn's disease	42	M	25.9	Stricturing	No	No
Patient 5	Crohn's disease	26	F	23.1	Stricturing	No	Yes, Ustekinumab
Patient 6	Crohn's disease	36	M	20	Stricturing	No	Yes, Ustekinumab
Patient 7	Crohn's disease	40	F	20.4	Inflammatory	Yes	No
Patient 8	Crohn's disease	66	F	28	Inflammatory	Yes	No
Patient 9	Control	51	M	32.9	N/A	No	No
Patient 10	Control	33	M	23.1	N/A	No	No
Patient 11	Control	49	F	47.1	N/A	No	No
Patient 12	Control	62	F	25.4	N/A	No	No

### 2.3 MSC adipogenesis and Oil‐Red‐O staining

Basal MSC medium consisted of DMEM: F12 plus 10% FBS, 1% L‐glutamine (200 mM) and 1% Penicillin–Streptomycin (10,000 U/ml) (Media Core, Cleveland Clinic, Cleveland, OH). 1 μM Dexamethasone (Millipore Sigma), 10 μM Insulin (Millipore Sigma), 0.5 mM IBMX (Millipore Sigma) and 5 μM Indomethacin (Millipore Sigma) were added for adipocyte differentiation medium. Subcutaneous and mesentery‐derived MSCs from one control and one CD patient were cultured in basal MSC medium (undifferentiated) or adipocyte differentiation medium. MSCs were differentiated for 10 days, with media changes every 3–4 days. Undifferentiated MSCs were assayed at 80% confluency. Lipid droplet staining was performed with Lipid (Oil Red O) Staining Kit's (Abcam, CA) protocol.

### 2.4 MSC surface marker expression and capacities tests

Flow cytometry was performed to assess protein surface marker expression. Isolated MSCs were washed and incubated with respective MSC surface marker antibodies (2 ug/ml anti‐CD45 IgG, anti‐CD34 IgG, anti‐CD14 IgG, anti‐CD44 IgG, anti‐CD90 IgG and anti‐CD105) or isotype control purchased from BioLegend (San Diego, CA), followed by flow cytometry analysis on a FACS Calibur flow cytometer (BD Biosciences). Senescence was determined with intracellular anti‐pSTAT3 staining (BioLegend). Apoptosis was determined with an Annexin V detection kit (BioLegend) and intracellular pSTAT3 staining according to manufacturer's protocol. Proliferation assay was performed with a BrdU ELISA kit (Roche) according to manufacturer's protocol.

### 2.5 Extracellular vesicle isolation

To prepare the EVs from the same MSC‐free medium, the previous listed medium(s) was ultra‐centrifuged for 16 h at 100,000 × *g* at 4°C in a 45Ti fixed angle rotor using polycarbonate tubes (Beckman Coulter, Brea, CA). After ultracentrifugation, the top layer medium suspension was harvested, filtered with a 0.22 μm PES filter and stored at 4°C. The EVs were extracted and concentrated from the culture media. Briefly, during the MSC harvest procedure, the cultured media was collected and filtered through a 0.22 μm filter to remove cell debris and large vesicles, followed by ultracentrifugation at 30,000 × *g* for 20 min to pellet larger microvesicles. The supernatants were then subjected to ultracentrifugation at 120,000 × *g* for 3 h to sediment the EVs. The resulting pellets were resuspended in PBS. Supernatant was discarded, and the EV pellet was resuspended in 200 μl 0.1 μM filtered PBS. For the TEM sample, 50 μl of EV suspension was mixed 1:1 with 4% 0.1 μM filtered PFA in PBS and stored at 4°C until processing. Remaining 50ul aliquots were stored at −80°C. Transmission electron microscopy (TEM) sample processing and imaging was performed by the Cleveland Clinic Imaging Core (Cleveland, Ohio) on 2% PFA‐fixed EVs. Zetaview analysis was performed by the Cleveland Clinic Flow Cytometry Core (Cleveland, Ohio).

### 2.6 RNA extraction and NGS

RNA was isolated from matched mesentery (Mes) and subcutaneous (SubQ) adipose tissues from three patients with either diverticulitis (Healthy) or Crohn's disease (CD) via Qiagen's RNeasy Plant Kit with DNase treatment according to manufacturer's protocol. RNA was submitted to the Case Western Reserve Genomics Core for NEBNext Ultra II Directional RNA library prep and 75 bp paired‐end NextSeq 550 High Output sequencing.

### 2.7 Bioinformatics

Bioinformatic analysis was performed by the Cleveland Institute for Computational Biology. Sequencing reads generated from the Illumina platform were assessed for quality and trimmed for adapter sequences using TrimGalore! v0.4.2 (Babraham Bioinformatics), a wrapper script for FastQC and cutadapt. Reads that passed quality control were then aligned to the human reference genome (GRCh37) using the STAR aligner v2.5.1. The alignment for the sequences was guided using the GENCODE annotation for hg19. The aligned reads were analysed for differential expression using Cufflinks v2.2.1, a RNASeq analysis package which reports the fragments per kilobase of exon per million fragments mapped (FPKM) for each gene. Differential analysis report was generated using Cuffdiff. Differential genes were identified using a significance cut‐off of q‐value <0.05.

Differential gene expression was analysed for 6 group comparisons: CD SubQ vs CD Mes, Healthy SubQ vs Healthy Mes, Healthy SubQ vs CD SubQ, Healthy Mes vs CD Mes, Healthy SubQ vs CD Mes, Healthy Mes vs CD SubQ.

STAR Aligner:

Dobin A, Davis CA, Schlesinger F, Drenkow J, Zaleski C, Jha S, Batut P, Chaisson M, Gingeras TR. STAR: ultrafast universal RNA‐seq aligner. *Bioinformatics*. 2013 Jan 1;29^1^:15–21. doi: 10.1093/bioinformatics/bts635. Epub 2012 Oct 25. PubMed PMID: 23104886; PubMed Central PMCID: PMC3530905.

Cufflinks:

Trapnell C, Williams BA, Pertea G, Mortazavi A, Kwan G, van Baren MJ, Salzberg SL, Wold BJ, Pachter L. Transcript assembly and quantification by RNA‐Seq reveals unannotated transcripts and isoform switching during cell differentiation. *NatBiotechnol*. 2010 May;28^5^:511–5. doi: 10.1038/nbt.1621. Epub 2010 May 2. PubMed PMID: 20436464; PubMed Central PMCID: PMC3146043.

### 2.8 Pathway analysis

Pathway analysis was performed with Qiagen's Ingenuity Pathway Analysis (IPA) software. The differentially expressed gene output was uploaded into IPA and core analysis was run. Finally, we focused the results related to T‐cell ‘Diseases and Functions’ and ‘Canonical Pathway’ outputs for analysis.

### 2.9 Murine model

Wild‐type (WT) mice (C57/Bl6 background) were maintained under pathogen‐free conditions in the animal facility of Lerner Research Institute, Cleveland Clinic, Cleveland OH. All procedures involving mice were approved by the Institutional Animal Care and Use Committee of Cleveland Clinic, and all were done in accordance with the US Department of Health and Human Services Guide for the Care and Use of Laboratory Animals and institutional guidelines.

### 2.10 Dextran sulphate sodium (DSS)‐induced colitis

Dextran sulphate sodium (DSS)‐induced Colitis has been widely used as an experimental model to study pathogenic mechanisms underlying IBD.^38^, ^39^ In brief, on Day 0, 3% DSS (MW 40 kDa; Sigma‐Aldrich, St. Louis, MO) was added to the drinking water. Previously published literature described that the mice would develop disease on Days 3–5.^38^, ^39^ Therefore, the delivery of MSCs isolated from healthy donors' subcutaneous adipose tissue (Normal), CD patients' subcutaneous adipose tissue (SubQ) and CD patients' mesenteric adipose tissue (Mesentery) (∼1 million cells per mouse), EVs from the aforementioned locations (∼1 million cells production per mouse), or PBS (control) was performed on Day 4. To grade the clinical severity of DSS‐induced colitis, mice were assessed for body weight (daily), stool consistency (daily) and hematochezia by faecal occult blood test (every other day) with clinical scores as shown in Table 2. Six days after injection (10 days from initiation of DSS), the mice were sacrificed, blood collected, and colon tissues were harvested and prepared for subsequent experiments. Colon lengths were measured, and sections submitted for H&E staining and histologic score evaluated.^40^

**TABLE 2:** Disease activity index scoring chart

Clinical score	Weight loss (%)	Stool consistency	Hematochezia
0	None	Normal	None
1	1–10%	Soft stool	Hemaoccult positive
2	10–20%	Diarrhoea	Gross blood
3	20%	Diarrhoea	n/a

### 2.11 Mouse T‐cell isolation and T‐cell flow cytometry

Naive or DSS‐treated mice were euthanized, and spleens were collected. Splenic tissue was compressed through a 100 μm cell strainer with 15 ml isolation buffer (PBS supplemented with 0.1% BSA and 2 mM EDTA). Cells were centrifuged at 350 *g* for 5 min followed by RBCs lysis on ice for 5 min with 10 ml 1X RBC Lysis Buffer (Biolegend, CA). Lysis buffer was diluted with 30 ml isolation buffer and centrifuged at 350 *g* for 5 min. Cell pellet was washed with 15 ml isolation buffer, centrifuged at 350 *g* for 5 min and resuspended in 1 ml for cell counts using Countess II (Invitrogen, MA). T cells were purified with Dynabeads® FlowComp™ Mouse Pan T (CD90.2) kit according to manufacturer's protocol (Invitrogen). T cells were counted and used for Flow Cytometry. Cells were blocked with TruStain FCx Plus antibody (Biolegend), and True‐Nuclear™ Transcription Factor Buffer Set (Biolegend) was used for fixation according to manufacturers’ protocol. Cell markers CD4, IL‐17A and IFN‐γ were purchased from Biolegend and flow performed on the Cytek Aurora (Cytek Biosciences).

### 2.12 Mouse Serum and colon homogenate preparation and multiplex assays

Mice were euthanized, and colon and sera were collected as previous described. Colons were snap‐frozen in liquid nitrogen and stored at −80°C until processed. Each colon was thawed on ice and quickly homogenized on ice in 1–2 ml of PBS containing a tablet of proteinase inhibitors (10 ml PBS/tablet; Millipore Sigma). Homogenized tissues were centrifuged at 2000 *g* at 4°C for 10 min, and then filtered, aliquoted and stored at −80°C until analysed by enzyme‐linked immunosorbent assay (ELISA). Protein concentrations were determined using a BCA Protein Assay kit (ThermoFisher, MA), and the concentrations of different mouse cytokines/chemokines in the homogenates or serums were measured by a multiplex assay (Eve Technologies) and normalized against the total protein of each tissue lysate or serum.

### 2.13 Isolation of infiltrated cells

After euthanization and tissue collection, the colon was washed by 4°C RPMI media (Invitrogen, MA). The colon was transferred to 10 ml fresh, cold RPMI and homogenized with 5 strokes in a tissue sonicator (Wheaton). The resulting cell suspension was gravity sieved through a 40 μm strainer (Invitrogen) to remove large aggregates. Cells were pelleted by centrifugation at 1000 *g* for 5 min at 4°C, and the supernatant was discarded. The cells were resuspended in 1 ml 70% Percoll (GE biotech, US) prepared in PBS, and overlaid with 1 ml 35% Percoll prepared in PBS. This discontinuous gradient was centrifuged at 800 *g* for 20 min at 4°C with braking disengaged to prevent disruption of the gradient interface layer. The central interface layer was collected and washed by cold RPMI. The cell suspension was pelleted by centrifugation at 1000 *g* for 5 min at 4°C, and the supernatant was discarded. The cell pellet was either lysed for isolation or processed for flow cytometry.

### 2.14 In Vitro Th1, Th2, Th9, Th17 and treg polarization assays

CD4^+^ T cells were isolated from WT mice by negative selection with EasySep Mouse CD4^+^ T Cell Isolation Kit (STEMCELL Technologies, Vancouver, Canada) and then cultured under Th1, Th2, Th9, Th17 and Treg polarizing conditions by following published protocols.^41^, ^42^ In brief, CD4^+^ T cells (2 × 10^4^ cells per well) were activated with plate‐bound 5 μg/ml anti‐mouse CD3 and 1 μg/ml anti‐CD28 (BioLegend) and then cultured at 37°C for 5 days. T‐cell differentiation was performed with the proteins and antibodies listed in Table S2. Finally, differentiated Th1, Th2, Th9, Th17 and Treg cells (day 5) were quantitated by intracellular staining of IFN‐γ, IL‐4, IL‐9, IL‐17 and Foxp3 (Biolegend, CA). Anti‐IL‐4 and anti‐IFN‐γ mouse antibodies were purchased from BioLegend (San Diego, CA). Recombinant mouse IL‐2, IL‐12, IL‐6 and TGF‐β proteins were purchased from PeproTech (Rocky Hill, NJ).

### 2.15 T‐cell inhibition assays

A conventional carboxyfluorescein succinimidyl ester (CFSE)–based T‐cell proliferation assay was used to test the T‐cell inhibitory activity of MSCs using previously described methods, with minor modifications.^43^ For mouse‐activated T‐cell proliferation assays, naive C57BL/6 mouse spleen cells were first enriched using nylon wool columns and labelled by incubating them with 0.3 μM of CFSE (Invitrogen, Carlsbad, CA) at 37°C for 8 min. After washing, 2.0 μg/ml of anti‐CD3 mAb and anti‐CD28 mAb (BD Biosciences) was added to the CFSE‐labelled spleen cells to activate T cells. The CFSE‐labelled, anti‐CD3/CD28 mAb‐activated cells were then aliquoted into wells of a 96‐well plate at a concentration of 0.4 × 10^6^ cells/well and incubated with different MSC/EV adipose sources in triplicate (healthy donor subcutaneous adipose tissue, CD subcutaneous adipose tissue and CD mesenteric adipose tissue derived). After 3 days of incubation, MSC/EV‐mediated T‐cell inhibition was assessed by measuring the proliferation of both CD4^+^/CD8^+^ T cells and the production of IFN‐γ following protocols described previously,^42^, ^44^ and by checking under a microscope the numbers and sizes of cell clusters formed by the proliferating cells. An aliquot of the T cells, following culture, was examined for viability by trypan blue exclusion method as described earlier.^28^

### 2.16 Human or mouse cytokines ELISAs

IL‐12p40, IL‐6 and IL‐10 levels in the MSC culture supernatants, IFN‐g, IL‐4, IL‐9, IL‐17a and IL‐10 were measured by standard ELISAs following manufacturer‐provided protocols (Biolegend).

### 2.17 Statistical analysis

Experiments were performed in triplicate. To determine whether statistically significant differences existed between groups, clinical scores were analysed by GraphPad Prism 8 (GraphPad Software, Inc.). One‐way analysis of variance (anova) and Dunnett's multiple comparison test were used to determine the significant differences. Probability (*p*) values less than 0.05 were considered statistically significant.

## 3 RESULTS

### 3.1 Surface marker expression and adipose differentiation capacity of the tissue‐derived MSCs are similar

**FIGURE 1:**
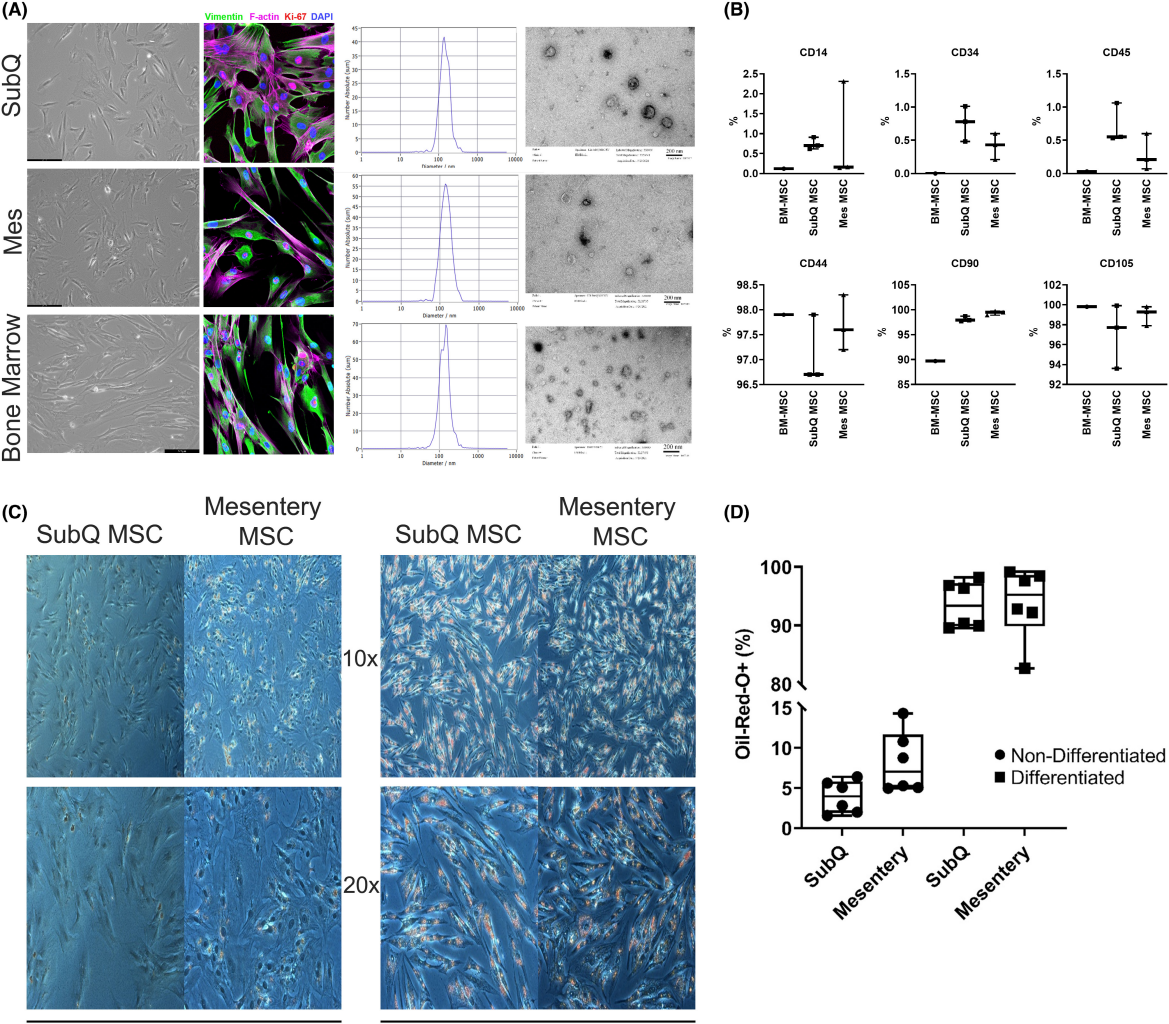
Characterization of BM‐MSCs, CD SubQ and CD Mes MSCs and EVs. (A) Bright field images and fluorescent staining of Vimentin (green), F‐Actin (pink) and KI‐67 (red) with DAPI (blue) counterstain of SubQ, Mes and Bone‐marrow derived MSCs; SubQ, Mes and BM‐MSC isolated EVs Zetaview and TEM images. (B) Quantification of cell surface markers CD14, CD34, CD45, CD44, CD90 and CD105 in SubQ, Mes and bone‐marrow derived MSCs. (C) Representative 10x and 20x Oil‐red‐O images of naïve and adipocyte differentiated SubQ and Mes MSCs (D) Quantification of Oil‐red‐O staining (*n* = 3). Data were presented as mean ± SEM

Crohn's subcutaneous and mesentery MSCs and EVs were compared to GMP‐grade bone‐marrow‐derived MSCs for characterization. There were no significant differences in the expression level of mesenchymal (Vimentin) and proliferation (KI‐67) surface makers between cell lines (Figure 1A, *p* > 0.05). On examination of intrinsic MSC markers CD14, CD34, CD44, CD90 and CD105, and the haematopoietic stem cell marker CD45, on MSCs by flow cytometry, expected cell surface markers were expressed and CD45 was not expressed (Figure 1B). In addition, MSCs from both subcutaneous and mesenteric adipose tissues differentiated into adipocytes as seen by the expected appearance of intracellular lipid droplets.^26^, ^45^, ^46^ (Figure 1C,D). EV particles also displayed similar size and morphology profiles (Figure 1A).

### 3.2 RNA sequencing of inflamed Crohn's disease mesentery tissue reveals an enrichment of T‐cell activation compared to non‐inflamed subcutaneous tissue

**FIGURE 2:**
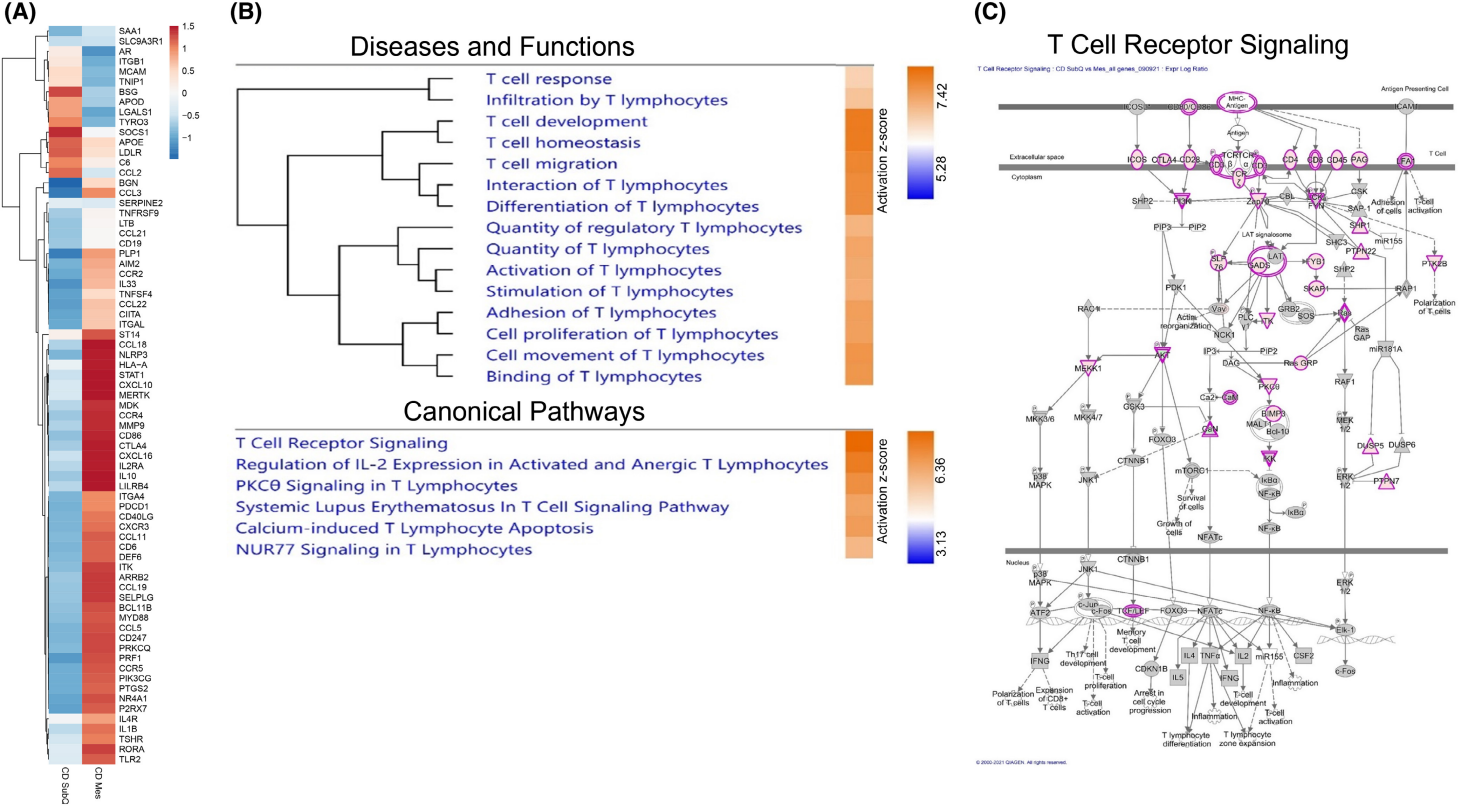
RNA sequencing results on T‐cell functions and pathways of CD SubQ vs CD Mes (*n* = 3). (A) Normalized FPKM z‐score heat map of differentially expressed genes in for the function ‘Infiltration of T Cells’. (B) Activation z‐score heat map of selected ‘Diseases and Functions’ and ‘Canonical Pathways’. (C) Canonical pathway of T‐cell receptor signalling. CD SubQ vs CD Mes differentially expressed genes highlighted in pink. Node colours represent fold change. Red: upregulated, Green: downregulated and Grey: expressed

RNA was isolated from three Crohn's disease (CD) patients' matched non‐inflamed SubQ and inflamed mesentery (Mes) adipose tissues and total RNA sequencing performed. We identified 1507 differentially expressed genes (DEG) between CD SubQ and CD Mes, of which 954 were associated with T cells. Figure 2A shows the normalized FPKM z‐scores of 211 DEG related to the infiltration of T lymphocytes which showed that the overall expression profile between CD SubQ and CD Mes was inversed. A pathway analysis with Qiagen's Ingenuity Pathway Analysis (IPA) software was performed to better understand the relationship between the DEG genes. A total of 15 diseases and functions and 6 canonical pathways related to T cells were significantly activated in CD Mes as compared to CD SubQ (Figure 2B and Table S1). T‐cell receptor signalling was the most significantly activated pathway in CD SubQ vs CD Mes, with 74 DEG present and a z‐score of 6.36 (Figure 2C). Consistent with the previous data, CD4 and CD8A genes were upregulated in CD Mes as compared to CD SubQ (1.65 and 3.53, respectively, q‐value <0.0001). IPA identified IL‐2 and IL‐6 as significantly activated upstream regulators in CD Mes compared to CD SubQ. IPA additionally identified activation of regulators IFN‐γ, IL1A, IL1B, IL4, IL12, GM‐CSF and TNF, which were not seen in the murine model.

### 3.3 Comparison of the self‐renewal and proliferation capacity of MSCs, the secreted soluble factors mediating MSC‐dependent immune regulation

**FIGURE 3:**
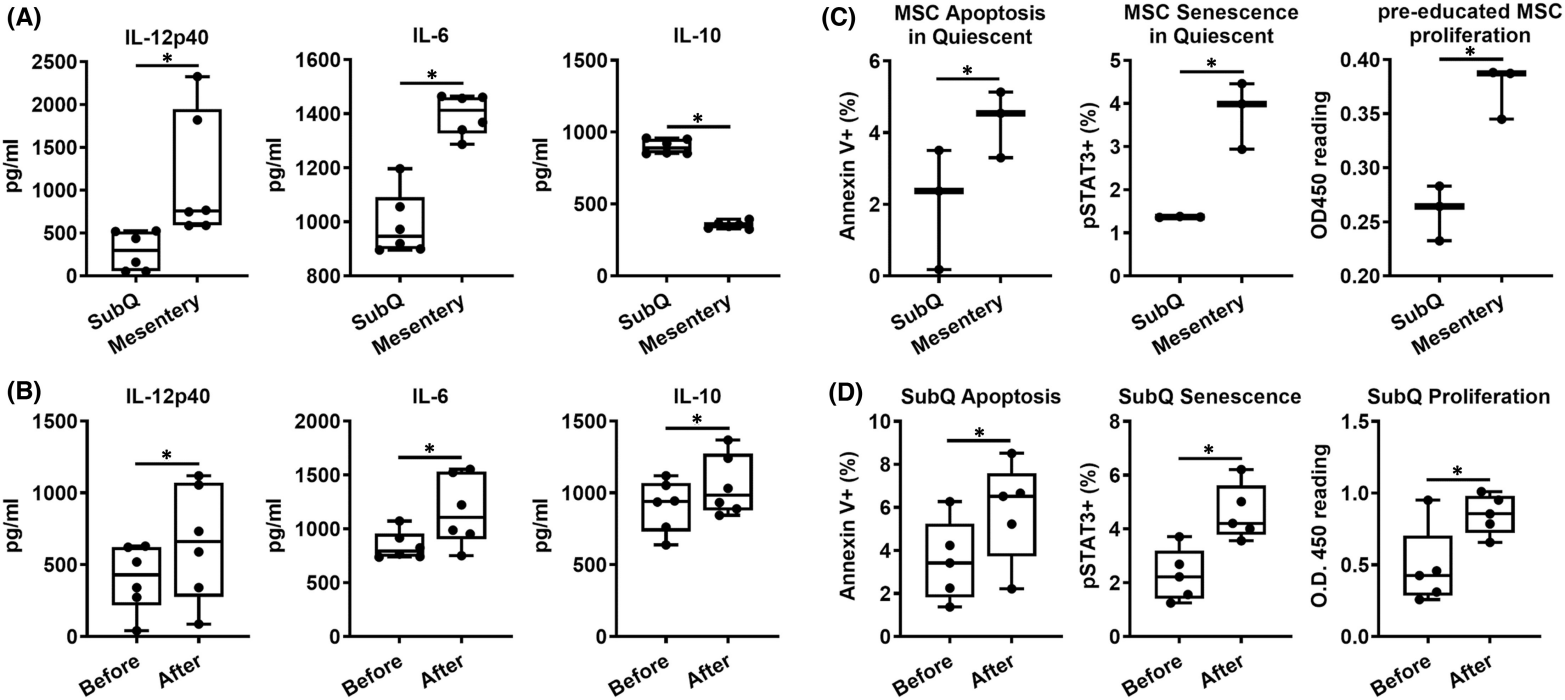
Secreted soluble factors, self‐renewal and proliferation capacity of MSCs. (A) Cytokine panels from cultured SubQ and mesentery MSC supernatants were measured for cytokine concentrations by ELISA (*n* = 6). (B) SubQ MSC cytokine levels before and after IFN‐γ and IL‐17 stimulation (*n* = 6). (C) SubQ and mesentery MSC measurements of apoptosis (Annexin V+) senescence (pSTAT3+) and proliferation (BrdU incorporation assay) (*n* = 3). (D) Measurements of apoptosis (Annexin V+) senescence (pSTAT3+) and proliferation (BrdU incorporation assay) before and after IFN‐γ and IL‐17 stimulation of SubQ MSCs (*n* = 5). Data were presented as mean ± SEM, one‐way anova * *p* < 0.05

Current studies have demonstrated that several factors contribute to MSC‐mediated effects on inflammation. MSCs constitutively, or upon stimulation, secrete large amounts of soluble factors closely associated with IBD progression, such as interleukin (IL)‐6, IL‐10 and IL‐12.^47^, ^48^, ^49^ IFN‐γ (Th1) and IL‐17 (Th17) are important inflammatory cytokines secreted by T cells in CD,^50^, ^51^, ^52^, ^53^ thereby interfering with the anti‐inflammatory activity of MSCs.^54^, ^55^ Subcutaneous and mesentery‐derived MSCs were tested for the production of cytokines IL‐6, IL‐10 and IL12p40. Mesenteric derived MSC secrete more inflammatory (IL‐6, IL12p40) and less anti‐inflammatory (IL‐10) cytokines (Figure 3A). To test the effect of IFN‐γ and IL‐17 on MSCs, we first stimulated subcutaneous derived MSCs with 100 U IFN‐γ and 100 U IL‐17 over 48 h and then assessed cytokine levels by ELISA. Consistent with mesenteric derived MSCs, stimulated subcutaneous MSCs secreted increased IL‐6 and IL12p40 after incubation with IFN‐γ and IL‐17 (Figure 3B), suggesting that IBD‐associated inflammatory cytokines IFN‐γ and IL‐17 upregulate cytokine secretion of MSCs.

Proliferation, apoptosis and senescence can be used to demonstrate cell function. MSCs show strong proliferative capacity according to the reported growth and doubling times.^56^ As detected by BrdU incorporation assay, mesenteric derived MSCs had a faster proliferation rate compared to SubQ MSCs without external stimuli under normal culture conditions (Figure 3C). Moreover, mesenteric derived MSCs underwent significantly increased apoptosis (Annexin V+) and more senescence (pSTAT3+) than SubQ‐derived MSCs (Figure 3C). In addition to basal functions, the SubQ MSCs were tested under 100 U IFN‐γ and 100 U IL‐17 stimulated conditions. In this pro‐inflammatory condition, ‘activated’ MSCs had significantly increased proliferation, apoptosis and senescence (Figure 3D).

### 3.4 Crohn's mesentery adipose tissue‐derived MSC treatment failed to attenuate murine DSS‐induced colitis

**FIGURE 4:**
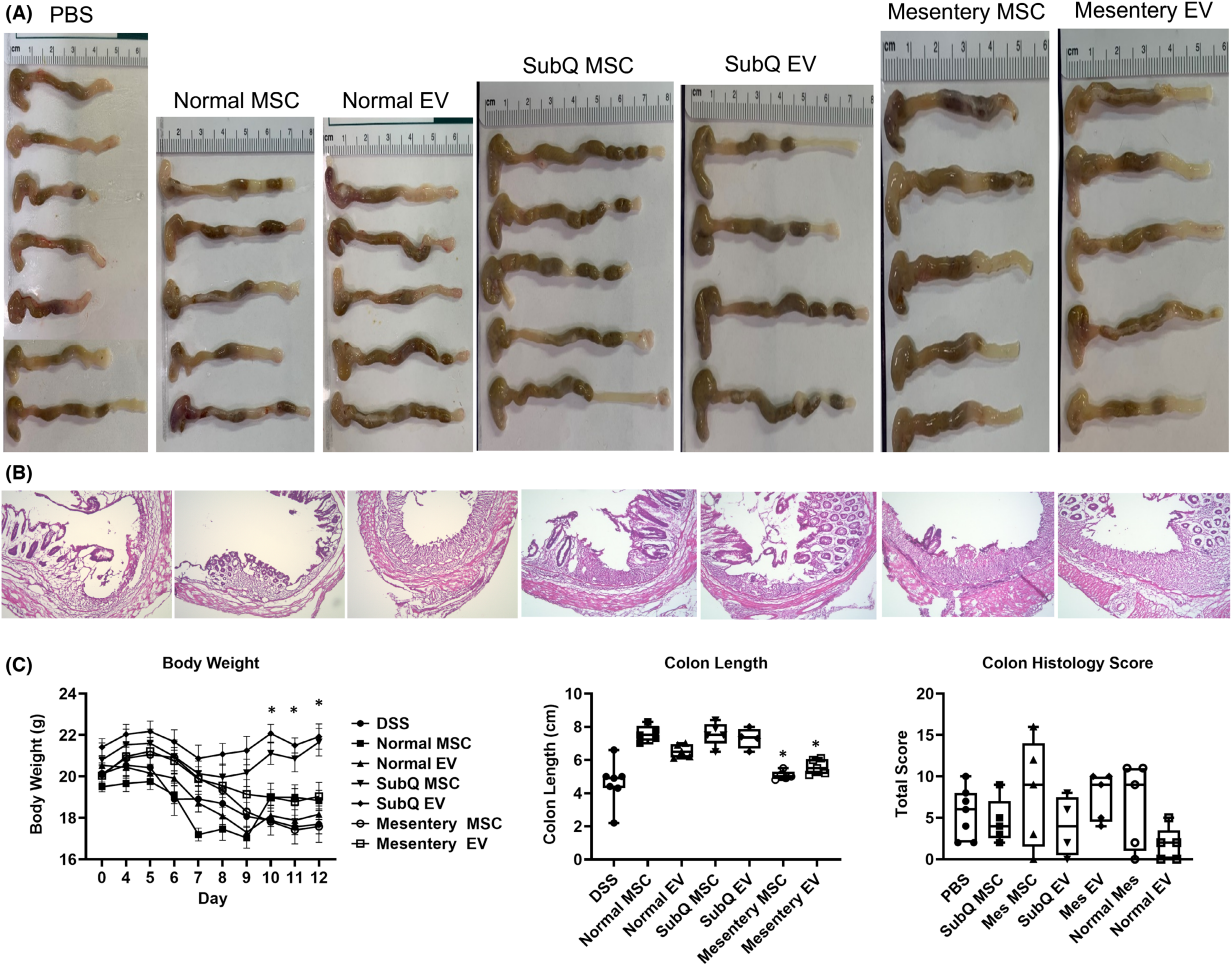
Anatomical analysis of DSS‐induced colitis mice treated with Normal, SubQ or mesentery MSCs or EVs. (A) Images of mouse colons, dissected from cecum to anus. (B) Colon H&E staining. (C) Body weight measurements over time; colon length and histology scores. Combined results from two experiments, *n* = 10. Data are represented as Mean ± SEM, *p < 0.05

Previous studies have demonstrated that MSCs or EVs are effective on T‐cell‐mediated diseases, such as multiple sclerosis and colitis^27^, ^28^, ^42^, ^44^ but the precise role of healthy or diseased MSCs or EVs in the pathogenesis of colitis remains unclear. To address this issue, colitis was induced in wild‐type mice (C57Bl6 background) by adding 3% DSS to the drinking water and colitis progression was assessed by monitoring clinical scores daily. Splenic CD4^+^/IL17A^+^ and CD4^+^/IFN‐γ^+^ T cells were decreased in DDS‐treated mice compared to naive water mice confirming an inflammatory response and T‐cell dysregulation in our model (Figure S1). Once the mice began to show mild clinical signs of colitis, they were treated with human subcutaneous or Crohn's disease mesentery MSCs (∼1 million cells per mouse), EVs (∼1 million cells production per mouse), or with PBS (control) by intraperitoneal (i.p.) injection and monitored the mice daily for 6 days. The disease activity index score at sacrifice was significantly reduced in both the normal and subcutaneous derived MSCs and EV‐treated mice compared to control PBS dosed mice but not in the diseased mesentery MSCs or EVs (Table 3). Immunological assays were performed as described. Treatment of DSS‐induced colitis with Crohn's disease mesentery‐derived MSCs/EVs, and control PBS, resulted in colitis progression, whereas in both normal and subcutaneous MSCs and its extracted EV‐treated mice the disease was attenuated. Subcutaneous MSC‐ and EV‐treated mice showed anatomical evidence, including weight gain and longer colon lengths, of diminished colitis 6 days after treatment (Figure 4A,C). Subcutaneous derived MSCs and EVs showed equivalent efficacy in treating DSS‐developed severe colitis (Figure 4).

**TABLE 3:** Disease activity index score for DSS‐induced colitis mice treated with normal, SubQ or Mes MSCs or EVs

Group	D0	D5	D6	D7	D9	D10
PBS	0	0.75 ± 0.26	1.31 ± 0.52	2.65 ± 0.15	2.88 ± 0.37	2.99 ± 0.14
SubQ MSC	0	0.18 ± 0.09 ^a^	0.49 ± 0.17 ^a^	0.75 ± 0.3 ^a^	1.11 ± 0.25 ^a^	1.64 ± 0.37 ^a^
SubQ EV	0	0.21 ± 0.08 ^a^	0.54 ± 0.13 ^a^	0.71 ± 0.28 ^a^	1.22 ± 0.24 ^a^	1.88 ± 0.65 ^a^
Mes MSC	0	0.71 ± 0.13 ^a^	0.98 ± 0.67 ^a^	1.96 ± 0.95 ^a^	2.78 ± 0.46 ^a^	2.95 ± 0.31 ^a^
Mes EV	0	0.59 ± 0.25 ^a^	1.22 ± 0.15 ^a^	2.21 ± 0.56 ^a^	2.92 ± 0.35 ^a^	2.97 ± 0.12 ^a^
Normal MSC	0	0.19 ± 0.12 ^a^	0.52 ± 0.27 ^a^	0.66 ± 0.42 ^a^	1.32 ± 0.41 ^a^	1.96 ± 0.41 ^a^
Normal EV	0	0.28 ± 0.11 ^a^	0.69 ± 0.22 ^a^	0.98 ± 0.22 ^a^	1.62 ± 0.69 ^a^	2.12 ± 0.72 ^a^

*Abbreviations*: D, day; MES, Mesentery; MSCs, Mesenchymal stem cells, EVs: Extracellular Vesicle; SubQ, subcutaneous.

*Note*: Values are presented as mean only or mean ± SEM. ^a^ *p* < 0.05.

### 3.5 Altered cell infiltration and cytokine production profiles in the colon of DSS mice after the different tissue‐derived MSC/EV therapy

**FIGURE 5:**
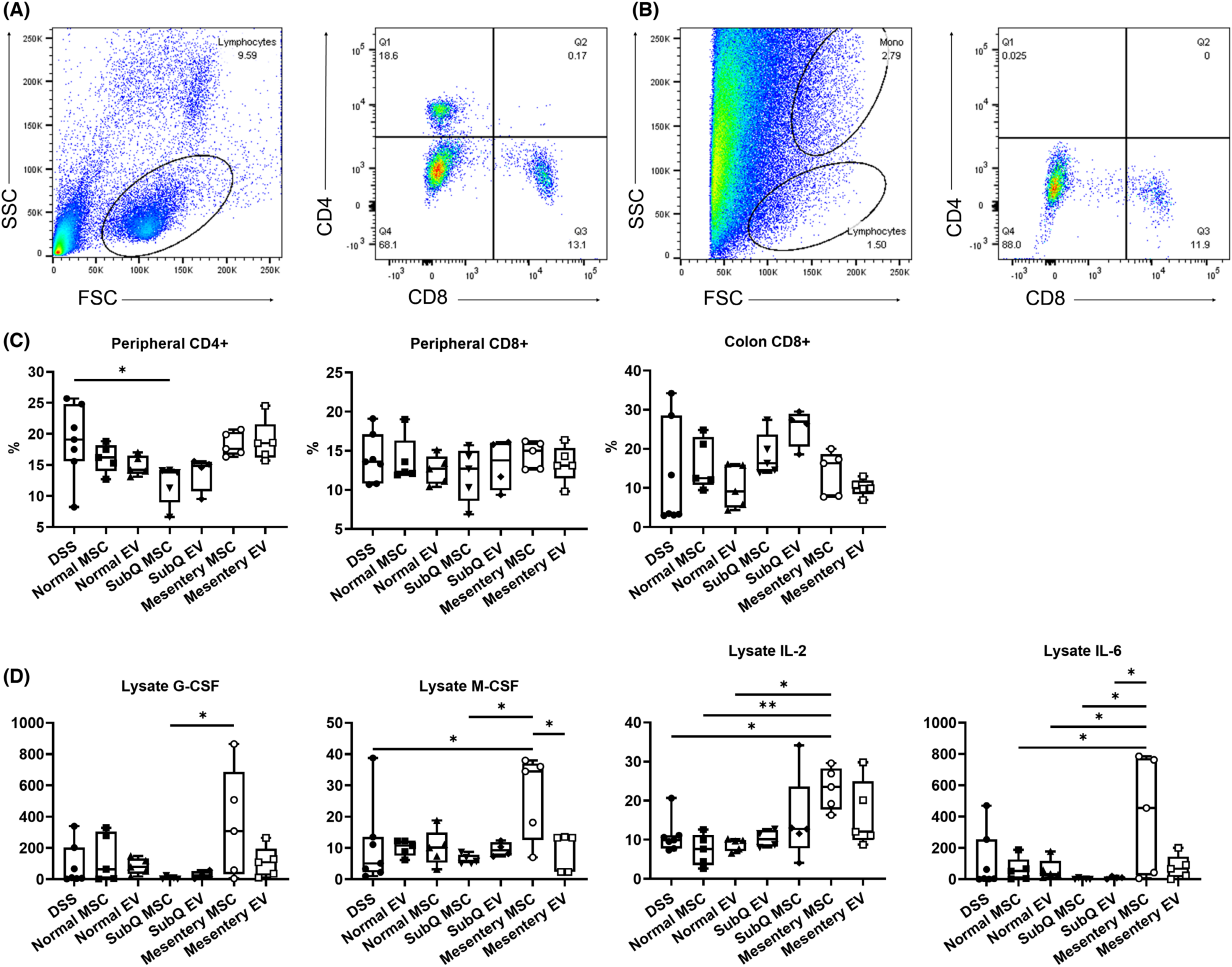
Analysis of CD4+ and CD8+ T cells and cytokine secretion in DDS‐induced colitis mice after Normal, SubQ or Mes MSC/EV treatment. Representative flow scatter plots of (A) peripheral blood and (B) local colon CD4+/CD8+ T cells with (C) cohort quantification. (D) Elisa measurement of G‐CSF, M‐CSF, IL‐2 and IL6 in colon homogenates. Symbols represent individual mouse colons (*n* = 4–7); bars show the mean ± SEM, one‐way anova * *p* < 0.05

The colon tissue in normal and SubQ MSC/EV‐treated mice exhibited leukocyte infiltration consistent with the significantly reduced clinical disease scores (Figure 5). In order to characterize the T effector cell phenotype in peripheral blood and colon isolated cells, the expression level of CD4 and CD8 was assessed. SubQ MSCs were able to decrease the number of peripheral CD4^+^ cells and CD8^+^ cells were identified as the primary T effector cell in the colon (Figure 5A–C). Using a cytokine multiplex assay, the levels of inflammatory cytokines from homogenates of collected colon tissues were measured and normalized against the concentrations of total protein (Figure 5D). Compared to the mesentery MSC group, the level of granulocyte colony‐stimulating factor (G‐CSF) was significantly lower in the SubQ MSC‐treated group and macrophage colony‐stimulating factor (M‐CSF) was significantly lower in the PBS and SubQ MSC‐treated groups. Furthermore, the mesentery MSC‐treated group had significantly lower IL‐2 in the PBS and Normal MSC/EV‐treated groups and IL‐6 was significantly lower in the Normal MSCs/EVs and SubQ MSC/EV‐treated groups. These results demonstrated that diseased mesentery tissue‐derived MSCs from Crohn's patients lose immunosuppressive capacity in the treatment of colitis, potentially by regulating pathogenic T‐cell responses and/or T‐cell infiltration into the colon (Figure 5, Figure S2).

### 3.6 Distinct MSC/EV regulation of T‐cell differentiation dependent on tissue source by in vitro assay

**FIGURE 6:**
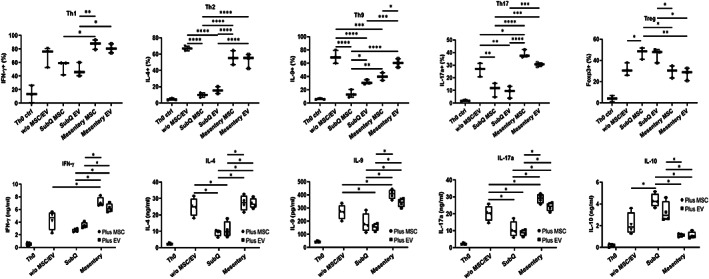
Intracellular staining and secretion of Th1, Th2, Th9, Th17 and Treg factors. Top) Intracellular staining and Bottom) cytokine secretion of IFN‐γ (Th1), IL‐4 (Th2), Il‐17A (Th17) and FoxP3 (Treg) in polarized murine T cells treated with SubQ or Mes MSCs/EVs (*n* = 4). Data are mean ± SEM, one‐way ANOVA * *p* < 0.05

To elucidate the mechanism by which diseased Crohn's mesentery tissue‐derived MSCs/EVs reduces pathogenic Th1/Th2/Th9/Th17/Treg responses and ameliorate disease severity in colitis, CD4^+^ T cells were isolated from naive WT mice and cultured under Th1/Th2/Th9/Th17/Treg polarization conditions. Flow cytometry analysis of intracellular IFN‐γ (Th1), IL‐4 (Th2), IL‐9 (Th9), IL‐17 (Th17) or FoxP3 (Treg) was performed. In these experiments, diseased mesentery tissue‐derived MSCs/EVs had impaired Th1/Th2/Th9/Th17/Treg development regulation compared with SubQ MSCs/ EVs (Figure 6).

### 3.7 Non‐inflamed microenvironment is important for MSCs/EVs to inhibit T cells

**FIGURE 7:**
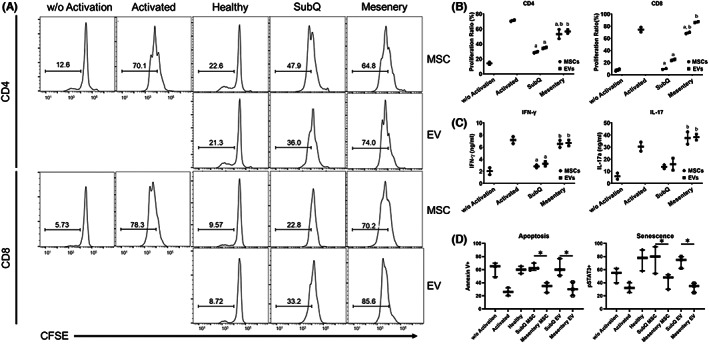
Proliferation, apoptosis, senescence and cytokine secretion of activated murine T cells after MSC/EV treatment. (A) Histogram of CD4 and CD8 proliferation of activated T cells co‐cultured with Normal, SubQ or Mes MSCs/EVs and (B) quantification. (C) IFN‐γ and IL‐17 secretion in media supernatants of activated T cells co‐cultured with SubQ or Mes MSCs/EVs. D) Apoptosis (Annexin V) and senescence (pSTAT3) of activated T cells co‐cultured with Normal, SubQ or Mes MSCs/EVs (*n* = 3). Data are mean ± SEM; one‐way ANOVA *, A, b = *p* < 0.05. (a: compared to activated, b: compared to SubQ MSCs and EVs).

MSCs are known to be an important immunosuppressive cell to inhibit T cells,^27^, ^28^, ^57^, ^58^ but whether the Crohn's induced pro‐inflammatory microenvironment contributes to the immunoregulatory activity of MSCs remains unclear. To clarify this issue, MSCs/EVs from subcutaneous adipose tissue or Crohn's mesentery adipose tissues were co‐cultured with isolated mouse T cells. Consistent with previous reports,^27^, ^28^ Crohn's mesentery MSCs/EVs were found to have reduced potency in both CD4 and CD8 T‐cell inhibition as compared with subcutaneous MSCs/EVs (Figure 7), suggesting that the local pro‐inflammatory environment results in the inability of MSCs/EVs to inhibit T cells. Compared with Crohn's mesentery MSCs or EVs, subcutaneous isolated MSCs and their EV products, inhibition of T‐cell proliferation and IFN‐γ, IL‐17a production significantly increased (Figure 7), suggesting a non‐inflamed microenvironment allows for T‐cell inhibition by MSCs/EVs.

### 3.8 Non‐inflamed microenvironment's MSCs/EVs reduce activated T‐cell survival

The Crohn's disease mesentery MSCs lost their inhibitory function and served as a negative regulator of T‐cell activation, consistent with the above results from the colitis studies that showed decreased Th1/Th17 responses. To further study this mechanism, we again activated mouse WT CD4^+^ T cells, treated with healthy donor MSCs, subcutaneous or mesentery MSCs/EVs and compared T‐cell apoptosis and senescence at 72 h by annexin V and pSTAT3 staining. In the presence of mesentery MSCs/EVs, mouse CD4 T cells underwent significantly more apoptosis (annexin V+ and pSTAT3+) than subcutaneous MSCs/EVs (Figure 7).

## 4 DISCUSSION

Despite an increasing incidence of CD in both developing and developed countries over the past four decades, the pathophysiology remains unknown.^59^, ^60^ Evidence suggests the pathophysiology is largely related to Crohn's specific immunopathogenic events.^31^, ^61^ Growing evidence suggests that hyperplastic mesenteric adipose tissue found at the sites of bowel wall damage, creeping fat, plays a role in the underlying disease progression. However, there have been varied suggestions as to how creeping fat plays an active role.^2^, ^3^ Mesenchymal stem cells (MSCs) are an active immunomodulatory cell which, when isolated from healthy adipose tissue, has demonstrated promising success in the treatment of certain Crohn's phenotypes.^62^ However, in local inflammatory conditions, MSCs may promote ongoing intestinal damage by contributing to dysregulated mechanistic function in T‐helper (Th) cells. Therefore, we investigated the influence of inflamed MSCs on T‐helper (Th) cells in an in vitro and in vivo model of CD. We found significant differences in T‐cell regulation among MSCs and EVs isolated from subcutaneous adipose tissue from healthy controls, subcutaneous adipose tissue from CD and Crohn's hyperplastic mesenteric tissue.

Initial characterization of our MSCs matched that of control BM‐MSCs, including canonical MSC markers CD44, CD90, and CD105, KI‐67 and vimentin staining, and adipogenic differentiation while at the same time lacking differentiated cell markers CD14, CD34 and CD45. Isolated EVs also matched in size and morphology. This confirmed that our MSC and EV populations were suitable for our studies.

Current publications have demonstrated that several factors contribute to MSC‐mediated effects on inflammation. MSCs constitutively, or upon stimulation, secrete large amounts of soluble factors closely associated with CD progression including interleukin (IL)‐6, IL‐10 and IL‐12.^47^, ^48^, ^49^ IFN‐γ (Th1) and IL‐17 (Th17) are important inflammatory cytokines secreted by T cells in CD,^50^, ^51^, ^52^, ^53^ thereby interfering with the anti‐inflammatory activity of MSCs.^54^, ^55^ When we compared inflammatory activity in vitro, Crohn's hyperplastic mesentery‐derived MSCs secreted significantly increased levels of inflammatory cytokines IL‐6 and IL12p40 and significantly decreased anti‐inflammatory cytokine IL‐10 compared to non‐inflamed MSCs, highlighting the pro‐inflammatory phenotype of MSCs derived from diseased Crohn's mesentery. We also found that if non‐inflammatory subcutaneous derived MSCs were exposed to pro‐inflammatory cytokines (IFN‐γ and IL‐17) in vitro, their phenotype was driven to a pro‐inflammatory state as seen by increased secretion of IL‐6 and I‐12p40 levels while IL‐10 was increased in response to injury.^63^, ^64^ This suggests that, in clinical practice, arterial delivery of healthy MSCs to the hypertrophic diseased mesentery surrounding inflamed bowel in CD may result in worsening inflammation, as healthy MSCs may transition to a pro‐inflammatory phenotype.^65^ In addition to the pro‐inflammatory phenotype, we demonstrated that these ‘activated’ MSCs had significantly increased proliferation, apoptosis and senescence. In line with this finding, the inflamed mesentery‐derived MSCs also contributed to increased T‐cell proliferation, apoptosis and senescence. This would contribute to ongoing mesenteric hyperplasia and expanding creeping fat around diseased segments of Crohn's bowel, creating an even greater pro‐inflammatory local microenvironment and may explain how diseased mesentery contributes to ongoing bowel wall damage and progression of CD.

In addition to the pro‐inflammatory phenotype identified in vitro, we found similar findings in our in vivo murine model of DDS‐induced colitis. Previous studies have demonstrated that MSCs are effective in treating T‐cell‐mediated diseases, such as multiple sclerosis and colitis^27^, ^28^, ^42^, ^44^ but the precise role of MSCs remains unclear. Using a DSS‐induced colitis murine model, we found that MSCs or EVs from Crohn's hyperplastic mesenteric adipose tissue did not attenuate disease progression. Increased disease activity index scores and colonic tissue perturbations compared to subcutaneous derived MSCs/EVs were significant in inflamed mesenteric MSCs or EVs. MSCs from Crohn's hyperplastic mesenteric adipose tissue resulted in a significantly increased local colonic secretion of inflammatory cytokines including granulocyte colony‐stimulating factor (G‐CSF), IL‐2, IL‐6 and macrophage colony‐stimulating factor (M‐CSF) than subcutaneous derived MSCs, pointing to an increase in leukocyte infiltration in the colon wall. Additionally, peripheral CD4^+^ cells were significantly or tended to be reduced under Normal and SubQ MSC/EV treatment. These results demonstrate that Crohn's hyperplastic diseased mesentery MSCs lose their immunosuppressive capacity in the treatment of colitis, potentially by regulating pathogenic T‐cell responses and/or T‐cell infiltration into the colon. Therefore, the local mesenteric environment has lost the ability to help restore healing, and is rather diseased, which may serve to promote worsening disease and ongoing inflammation.

RNA sequencing of non‐inflamed subcutaneous and matched inflamed mesentery CD tissue revealed multiple effects on T‐cell functions and pathways, such as an increase in T‐cell infiltration, including CD4^+^ and CD8^+^ T cells, and activation of T‐cell receptor signalling in the inflamed mesentery compared to non‐inflamed subcutaneous tissue. Additionally, inflammatory cytokines IFN‐γ, IL1A, IL1B, IL2, IL4, IL6 IL12, GM‐CSF and TNF were identified as upstream regulators of the differentially expressed gene set. These results support the pro‐inflammatory microenvironment and T‐cell dysregulation in diseased Crohn's mesentery tissue, corroborating our findings in our DSS‐induced colitis murine experiments under a human setting.

MSCs are known to be an important immunosuppressive cell to inhibit T cells,^27^, ^28^, ^57^, ^58^ but whether the Crohn's induced pro‐inflammatory microenvironment contributes to the immunoregulatory activity of MSCs remains unclear. To elucidate the mechanism by which hyperplastic Crohn's mesentery tissue‐derived MSCs alter T‐cell response, we co‐cultured MSCs and MSC‐derived EVs from subcutaneous adipose tissue or Crohn's mesentery adipose tissues with isolated mouse T cells. We found that the inflammatory environment of hyperplastic mesenteric tissue leads to impaired Th1, Th2, Th9, Th17 and Treg development as compared with subcutaneous derived MSCs and EVs. Intracellular staining and cytokine secretion of IFN‐γ (Th1), IL‐4 (Th2), Il‐17A (Th17) and FoxP3 (Treg) revealed that SubQ MSCs and EVs were able to significantly inhibit T‐cell activation while Mesentery MSCs and EVs were not. IL‐9 (Th9) showed similarly significant or trending results. In addition, consistent with previous reports,^27^, ^28^ we found that Crohn's hyperplastic mesentery‐derived MSCs and EVs had reduced potency in both CD4^+^ and CD8^+^ T‐cell inhibition as compared with subcutaneous MSCs and EVs as revealed by increased CD4^+^ and CD8^+^ T‐cell proliferation and decreased apoptosis and senescence. This suggests the local pro‐inflammatory environment results in the inability of MSCs to inhibit T cells. Thus, the ongoing pro‐inflammatory state of diseased hyperplastic mesentery in Crohn's disease may be partly driven by the loss in the local MSCs' inhibitory function to serve as a negative regulator of T‐cell activation.

To date, the precise mechanistic role of Th cells in inflammatory bowel disease remains unclear. However, it has been established that CD and ulcerative colitis (UC) have distinct Th effector cell cytokine expression profiles.^66^ This is critical as the associated cytokine loci regulate the development and function of intestinal Th cell subsets, such as IFN‐γ‐secreting Th1 cells, IL‐17‐secreting Th17 cells and Foxp3‐expressing T regulatory cells (Tregs).^67^ Inflammation in CD is generally associated with increased IFN‐γ and IL‐17A expression, whereas Th2 cytokines (IL‐4) predominate in UC.^68^, ^69^ On the contrary, T‐cell‐driven immune response predominates in CD, and most of the pathophysiological changes in CD inflamed tissue can be related to the effects of Th1 and Th17 cells.^30^, ^31^, ^32^, ^33^, ^34^, ^35^, ^36^, ^37^ Consistent with these findings, we found that Crohn's hyperplastic diseased mesentery tissue‐derived MSCs had impaired Th1/Th2/Th9/Th17/Treg development regulation compared with both healthy control and CD subcutaneous adipose MSCs. In addition, Crohn's hyperplastic diseased mesentery tissue‐derived MSCs had reduced potency in CD4 and CD8 T‐cell inhibition as compared to MSCs from subcutaneous tissue. In contrast, the non‐inflammatory environment of subcutaneous tissue in both Crohn's patients and normal healthy controls showed MSCs drove the inhibition of T‐cell proliferation. This highlights the potential mechanism by which MSCs contribute to a pro‐inflammatory state in hyperplastic mesenteric tissue and underscores the fact that the adipose tissue of the mesentery is its own organ and its pro‐inflammatory state does not always follow the state of the subcutaneous adipose tissue from a Crohn's patient. Thus, even though CD is systemic, the hyperplastic mesentery behaves quite differently from other sources of adipose tissue in a Crohn's patient. These results demonstrate that diseased mesentery tissue‐derived MSCs from Crohn's patients lose immunosuppressive capacity in the treatment of colitis, potentially by regulating pathogenic T‐cell responses and/or T‐cell infiltration into the colon.

Throughout our in vitro and in vivo studies, MSCs were compared to MSC‐derived extracellular vesicles (EVs), acellular membrane‐bound particles secreted from MSCs carrying protein, mRNA and miRNA. EVs can locally or systemically communicate with other cells and are thought to be the way MSCs carry out their paracrine functions.^70^, ^71^, ^72^, ^73^ Given the limitations of cost, scalability and delivery of MSCs, there has been increasing interest in EVs as a therapeutic. In fact, EVs have already demonstrated the ability to reverse acute kidney injury, vascular injury, pulmonary hypertension and obesity.^74^, ^75^, ^76^, ^77^, ^78^ Interestingly, we found that MSC‐derived EVs and MSCs were equivalent in our murine colitis model and also inhibited T‐cell proliferation in the same fashion as MSCs. This adds to the already increasing evidence that EVs alter cell‐to‐cell communication.^79^ If EVs are found to be equivalent to MSCs with regard to immunomodulatory effects and efficacy as a clinical therapeutic, future work could focus on engineering MSCs to produce an individualized EV product, specifically tailored to a particular need or disease state.^80^, ^81^, ^82^

There are limitations to this paper worth mentioning. First, the work was carried out by in vitro assays and by the established in vivo murine colitis model, which does not necessarily recapitulate the human disease. While our RNA‐seq results provide some human tissue‐derived evidence, whether this is translatable to human disease therapy remains unknown. Second, we are still uncertain as to whether inflammation in the bowel wall drives inflammation in the hyperplastic mesentery found in Crohn's disease, or the reverse with hyperplastic mesentery driving bowel wall damage. In all likelihood, this process is bidirectional with each contributing to the ongoing disease process, but this remains unknown. Third, additional immune cell subtypes including B cells, natural killer cells and macrophages likely play a significant role in CD, but these were not investigated within our study and will be interesting to consider for future investigations.

## 5 CONCLUSION

Diseased mesentery tissue‐derived MSCs from Crohn's patients lose immunosuppressive capacity in the treatment of colitis by regulating pathogenic T‐cell responses and/or T‐cell infiltration into the colon. In this way, the mesentery acts as an independent inflammatory organ abutting diseased bowel which may play a significant role in the pathophysiology of CD.

## SUMMARY

Crohn's mesentery is pathognomonic and referred to as ‘creeping fat’. How the mesentery plays a role in Crohn's remains unanswered. We found that mesenchymal stem cells within the mesentery propagate an inflammatory state.

## AUTHOR CONTRIBUTIONS

**Neda Dadgar:** Conceptualization (equal); data curation (equal); formal analysis (equal); funding acquisition (equal); investigation (equal); methodology (equal); project administration (equal); resources (equal); software (equal); supervision (equal); validation (equal); visualization (equal); writing – original draft (equal); writing – review and editing (equal). **Jessica Altemus:** Conceptualization (equal); data curation (equal); formal analysis (equal); funding acquisition (equal); investigation (equal); methodology (equal); project administration (equal). **Yan Li:** Conceptualization (equal); data curation (equal); formal analysis (equal); funding acquisition (equal); investigation (equal); methodology (equal); project administration (equal); resources (equal); software (equal); supervision (equal); validation (equal); visualization (equal); writing – original draft (equal). **Amy Lightner:** Conceptualization (equal); data curation (equal); formal analysis (equal); funding acquisition (equal); investigation (equal); methodology (equal); project administration (equal); resources (equal); software (equal); supervision (equal); validation (equal); visualization (equal); writing – original draft (equal); writing – review and editing (equal).

## CONFLICT OF INTEREST

Amy Lightner declares the consultant of Takeda. Other authors declare that there is no conflict of interest regarding the publication of this article.

## CONSENT TO PUBLISH

All the authors and the institution are consented to publish.

## Supporting information


Figure S1

Figure S2

Table S1–S2

## Data Availability

The date will be available according to request

## References

[1] Kredel LI , Siegmund B . Adipose‐tissue and intestinal inflammation ‐ visceral obesity and creeping fat. Front Immunol. 2014;5:462. 25309544 10.3389/fimmu.2014.00462 PMC4174117

[2] Ha CWY , Martin A , Sepich‐Poore GD , et al. Translocation of viable gut microbiota to mesenteric adipose drives formation of creeping fat in humans. Cell. 2020;183:666‐683.e17. 32991841 10.1016/j.cell.2020.09.009 PMC7521382

[3] Coffey CJ , Kiernan MG , Sahebally SM , et al. Inclusion of the mesentery in ileocolic resection for Crohn's disease is associated with reduced surgical recurrence. J Crohns Colitis. 2018;12(10):1139‐1150. 29309546 10.1093/ecco-jcc/jjx187 PMC6225977

[4] Rohban R , Pieber TR . Mesenchymal stem and progenitor cells in regeneration: tissue specificity and regenerative potential. Stem Cells Int. 2017;2017:5173732‐5173716. 28286525 10.1155/2017/5173732 PMC5327785

[5] Via AG , Frizziero A , Oliva F . Biological properties of mesenchymal stem cells from different sources. Muscles Ligaments Tendons J. 2012;2(3):154‐162. 23738292 PMC3666517

[6] Scarpone M , Kuebler D , Chambers A , et al. Isolation of clinically relevant concentrations of bone marrow mesenchymal stem cells without centrifugation. J Transl Med. 2019;17(1):10. 30611285 10.1186/s12967-018-1750-x PMC6321705

[7] Meppelink AM , Wang XH , Bradica G , et al. Rapid isolation of bone marrow mesenchymal stromal cells using integrated centrifuge‐based technology. Cytotherapy. 2016;18(6):729‐739. 27173749 10.1016/j.jcyt.2016.03.291

[8] Yi T , Kim SN , Lee HJ , et al. Manufacture of clinical‐grade human clonal mesenchymal stem cell products from single Colony forming unit‐derived colonies based on the subfractionation culturing method. Tissue Eng Part C Methods. 2015;21(12):1251‐1262. 26421757 10.1089/ten.TEC.2015.0017

[9] Rojewski MT , Fekete N , Baila S , et al. GMP‐compliant isolation and expansion of bone marrow‐derived MSCs in the closed, automated device quantum cell expansion system. Cell Transplant. 2013;22(11):1981‐2000. 23107560 10.3727/096368912X657990

[10] Berman DM , Willman MA , Han D , et al. Mesenchymal stem cells enhance allogeneic islet engraftment in nonhuman primates. Diabetes. 2010;59(10):2558‐2568. 20622174 10.2337/db10-0136 PMC3279532

[11] Ichiyanagi T , Anabuki K , Nishijima Y , Ono H . Isolation of mesenchymal stem cells from bone marrow wastes of spinal fusion procedure (TLIF) for low back pain patients and preparation of bone dusts for transplantable autologous bone graft with a serum glue. Biosci Trends. 2010;4(3):110‐118. 20592461

[12] Horn P , Bork S , Diehlmann A , et al. Isolation of human mesenchymal stromal cells is more efficient by red blood cell lysis. Cytotherapy. 2008;10(7):676‐685. 18985474 10.1080/14653240802398845

[13] Ryan ST , Hosseini‐Beheshti E , Afrose D , et al. Extracellular vesicles from mesenchymal stromal cells for the treatment of inflammation‐related conditions. Int J Mol Sci. 2021;22(6):3023. 33809632 10.3390/ijms22063023 PMC8002312

[14] Altemus J , Dadgar N , Li Y , Lightner AL . Adipose tissue‐derived mesenchymal stem cells' acellular product extracellular vesicles as a potential therapy for Crohn's disease. J Cell Physiol. 2022;237(7):3001‐3011. 35522572 10.1002/jcp.30756 PMC9544647

[15] Nazari H , Naei VY , Tabasi AH , et al. Advanced regenerative medicine strategies for treatment of perianal fistula in Crohn's disease. Inflamm Bowel Dis. 2022;28(1):133‐142. 34291798 10.1093/ibd/izab151

[16] Djouad F , Bouffi C , Ghannam S , Noel D , Jorgensen C . Mesenchymal stem cells: innovative therapeutic tools for rheumatic diseases. Nat Rev Rheumatol. 2009;5(7):392‐399. 19568253 10.1038/nrrheum.2009.104

[17] Ghannam S , Bouffi C , Djouad F , Jorgensen C , Noel D . Immunosuppression by mesenchymal stem cells: mechanisms and clinical applications. Stem Cell Res Ther. 2010;1(1):2. 20504283 10.1186/scrt2 PMC2873698

[18] Imam T , Park S , Kaplan MH , Olson MR . Effector T helper cell subsets in inflammatory bowel diseases. Front Immunol. 2018;9:1212. 29910812 10.3389/fimmu.2018.01212 PMC5992276

[19] Nikolakis D , de Voogd FA , Pruijt MJ , Grootjans J , van de Sande MG , D'Haens GR . The role of the lymphatic system in the pathogenesis and treatment of inflammatory bowel disease. Int J Mol Sci. 2022;23(3):1854. 35163775 10.3390/ijms23031854 PMC8836364

[20] Liao F , Lu X , Dong W . Exosomes derived from T regulatory cells relieve inflammatory bowel disease by transferring miR‐195a‐3p. IUBMB Life. 2020;72(12):2591‐2600. 10.1002/iub.2385 33108032

[21] Ocansey DK , Zhang L , Wang Y , et al. Exosome‐mediated effects and applications in inflammatory bowel disease. Biol Rev. 2020;95(5):1287‐1307. 32410383 10.1111/brv.12608 PMC7540363

[22] Valter M , Verstockt S , Finalet Ferreiro J , Cleynen I . Extracellular vesicles in inflammatory bowel disease: small particles, big players. Journal of Crohn's and Colitis. 2021;15(3):499‐510. 10.1093/ecco-jcc/jjaa179 32905585

[23] Akintoye SO , Lam T , Shi S , Brahim J , Collins MT , Robey PG . Skeletal site‐specific characterization of orofacial and iliac crest human bone marrow stromal cells in same individuals. Bone. 2006;38(6):758‐768. 16403496 10.1016/j.bone.2005.10.027

[24] Ackema KB , Charite J . Mesenchymal stem cells from different organs are characterized by distinct topographic Hox codes. Stem Cells Dev. 2008;17(5):979‐991. 18533811 10.1089/scd.2007.0220

[25] Igarashi A , Segoshi K , Sakai Y , et al. Selection of common markers for bone marrow stromal cells from various bones using real‐time RT‐PCR: effects of passage number and donor age. Tissue Eng. 2007;13(10):2405‐2417. 17596118 10.1089/ten.2006.0340

[26] Li Y , Lin F . Mesenchymal stem cells are injured by complement after their contact with serum. Blood. 2012;120(17):3436‐3443. 22966167 10.1182/blood-2012-03-420612 PMC3482856

[27] Li Y , Qiu W , Zhang L , Fung J , Lin F . Painting factor H onto mesenchymal stem cells protects the cells from complement‐ and neutrophil‐mediated damage. Biomaterials. 2016;102:209‐219. 27343468 10.1016/j.biomaterials.2016.05.055 PMC5290709

[28] Li Y , Fung J , Lin F . Local inhibition of complement improves mesenchymal stem cell viability and function after administration. Mol Ther. 2016;24(9):1665‐1674. 27377042 10.1038/mt.2016.142 PMC5113108

[29] Li Y , Lin F . Decoy nanoparticles bearing native C5a receptors as a new approach to inhibit complement‐mediated neutrophil activation. Acta Biomater. 2019;99:330‐338. 31446047 10.1016/j.actbio.2019.08.033 PMC7066532

[30] Ko JK , Auyeung KK . Inflammatory bowel disease: etiology, pathogenesis and current therapy. Curr Pharm des. 2014;20(7):1082‐1096. 23782147 10.2174/13816128113199990416

[31] Fiocchi C . Inflammatory bowel disease: etiology and pathogenesis. Gastroenterology. 1998;115(1):182‐205. 9649475 10.1016/s0016-5085(98)70381-6

[32] Kirsner JB . Inflammatory bowel disease. Considerations of etiology and pathogenesis. Am J Gastroenterol. 1978;69(3 Pt 1):253‐271. 665645

[33] Shorter RG , Huizenga KA , Spencer RJ . A working hypothesis for the etiology and pathogenesis of nonspecific inflammatory bowel disease. Am J Dig Dis. 1972;17(11):1024‐1032. 5082428 10.1007/BF02239143

[34] Strober W , Kelsall B , Fuss I , et al. Reciprocal IFN‐gamma and TGF‐beta responses regulate the occurrence of mucosal inflammation. Immunol Today. 1997;18(2):61‐64. 9057354 10.1016/s0167-5699(97)01000-1

[35] Fais S , Capobianchi MR , Pallone F , et al. Spontaneous release of interferon gamma by intestinal lamina propria lymphocytes in Crohn's disease. Kinetics of in vitro response to interferon gamma inducers. Gut. 1991;32(4):403‐407. 1902808 10.1136/gut.32.4.403 PMC1379080

[36] Auto‐immunity, allergy and inflammation. J Invest Dermatol. 2015;135(Suppl 2):S1‐S17. 10.1038/jid.2015.266 26269260

[37] Macdonald TT , Monteleone G . Immunity, inflammation, and allergy in the gut. Science. 2005;307(5717):1920‐1925. 15790845 10.1126/science.1106442

[38] Eichele DD , Kharbanda KK . Dextran sodium sulfate colitis murine model: an indispensable tool for advancing our understanding of inflammatory bowel diseases pathogenesis. World J Gastroenterol. 2017;23(33):6016‐6029. 28970718 10.3748/wjg.v23.i33.6016 PMC5597494

[39] Perse M , Cerar A . Dextran sodium sulphate colitis mouse model: traps and tricks. J Biomed Biotechnol. 2012;2012:718617. 22665990 10.1155/2012/718617 PMC3361365

[40] Rachmilewitz D , Karmeli F , Takabayashi K , Raz E . Amelioration of experimental colitis by probiotics is due to the immunostimulatory effects of its DNA. 2002. WB Saunders Co independence square west curtis center, STE 300, Philadelphia…. p A398‐A398.

[41] Harrington LE , Hatton RD , Mangan PR , et al. Interleukin 17‐producing CD4+ effector T cells develop via a lineage distinct from the T helper type 1 and 2 lineages. Nat Immunol. 2005;6(11):1123‐1132. 16200070 10.1038/ni1254

[42] Li Y , Singer NG , Whitbred J , Bowen MA , Fox DA , Lin F . CD6 as a potential target for treating multiple sclerosis. Proc Natl Acad Sci U S A. 2017;114(10):2687‐2692. 28209777 10.1073/pnas.1615253114 PMC5347585

[43] Tu Z , Li Y , Smith DS , et al. Retinal pericytes inhibit activated T cell proliferation. Invest Ophthalmol Vis Sci. 2011;52(12):9005‐9010. 22003106 10.1167/iovs.11-8008 PMC3231798

[44] Li Y , Kim BG , Qian S , et al. Hepatic stellate cells inhibit T cells through active TGF‐beta1 from a cell surface‐bound latent TGF‐beta1/GARP complex. J Immunol. 2015;195(6):2648‐2656. 26246140 10.4049/jimmunol.1500139 PMC4784714

[45] Kuhbier JW , Weyand B , Radtke C , Vogt PM , Kasper C , Reimers K . Isolation, characterization, differentiation, and application of adipose‐derived stem cells. Adv Biochem Eng Biotechnol. 2010;123:55‐105. 20091288 10.1007/10_2009_24

[46] Lu LL , Song YP , Wei XD , Fang BJ , Zhang YL , Li YF . Comparative characterization of mesenchymal stem cells from human umbilical cord tissue and bone marrow. Zhongguo Shi Yan Xue Ye Xue Za Zhi. 2008;16(1):140‐146. 18315918

[47] Haynesworth SE , Baber MA , Caplan AI . Cytokine expression by human marrow‐derived mesenchymal progenitor cells in vitro: effects of dexamethasone and IL‐1 alpha. J Cell Physiol. 1996;166(3):585‐592. 8600162 10.1002/(SICI)1097-4652(199603)166:3<585::AID-JCP13>3.0.CO;2-6

[48] Albini A , Melchiori A , Santi L , Liotta LA , Brown PD , Stetler‐Stevenson WG . Tumor cell invasion inhibited by TIMP‐2. J Natl Cancer Inst. 1991;83(11):775‐779. 1645772 10.1093/jnci/83.11.775

[49] Di Nicola M , Carlo‐Stella C , Magni M , et al. Human bone marrow stromal cells suppress T‐lymphocyte proliferation induced by cellular or nonspecific mitogenic stimuli. Blood. 2002;99(10):3838‐3843. 11986244 10.1182/blood.v99.10.3838

[50] Chen Z , Ni W , Yang C , et al. Therapeutic effect of Amomum villosum on inflammatory bowel disease in rats. Front Pharmacol. 2018;9:639. 29973876 10.3389/fphar.2018.00639 PMC6019447

[51] Schardey J , Globig AM , Janssen C , et al. Vitamin D inhibits pro‐inflammatory T cell function in patients with inflammatory bowel disease. J Crohns Colitis. 2019;13(12):1546‐1557. 31051495 10.1093/ecco-jcc/jjz090

[52] Balbas‐Martinez V , Ruiz‐Cerda L , Irurzun‐Arana I , et al. A systems pharmacology model for inflammatory bowel disease. PLoS One. 2018;13(3):e0192949. 29513758 10.1371/journal.pone.0192949 PMC5841748

[53] Triggianese P , Conigliaro P , Chimenti MS , et al. Evidence of IL‐17 producing innate lymphoid cells in peripheral blood from patients with enteropathic spondyloarthritis. Clin Exp Rheumatol. 2016;34(6):1085‐1093. 27782868

[54] da Costa GF , Paz AH . Cell membrane and bioactive factors derived from mesenchymal stromal cells: cell‐free based therapy for inflammatory bowel diseases. World J Stem Cells. 2019;11(9):618‐633. 31616539 10.4252/wjsc.v11.i9.618 PMC6789183

[55] Heidari M , Pouya S , Baghaei K , et al. The immunomodulatory effects of adipose‐derived mesenchymal stem cells and mesenchymal stem cells‐conditioned medium in chronic colitis. J Cell Physiol. 2018;233(11):8754‐8766. 29797577 10.1002/jcp.26765

[56] Yang J , Song T , Wu P , et al. Differentiation potential of human mesenchymal stem cells derived from adipose tissue and bone marrow to sinus node‐like cells. Mol Med Rep. 2012;5(1):108‐113. 21971826 10.3892/mmr.2011.611

[57] Haddad R , Saldanha‐Araujo F . Mechanisms of T‐cell immunosuppression by mesenchymal stromal cells: what do we know so far? Biomed Res Int. 2014;2014:216806. 25025040 10.1155/2014/216806 PMC4082893

[58] van Megen KM , van't Wout ET , Lages Motta J , Dekker B , Nikolic T , Roep BO . Activated mesenchymal stromal cells process and present antigens regulating adaptive immunity. Front Immunol. 2019;10:694. 31001285 10.3389/fimmu.2019.00694 PMC6457321

[59] Danese S , Fiocchi C . Ulcerative colitis. N Engl J Med. 2011;365(18):1713‐1725. 22047562 10.1056/NEJMra1102942

[60] Philippe D , Favre L , Foata F , et al. Bifidobacterium lactis attenuates onset of inflammation in a murine model of colitis. World J Gastroenterol. 2011;17(4):459‐469. 21274375 10.3748/wjg.v17.i4.459 PMC3027012

[61] Bouma G , Strober W . The immunological and genetic basis of inflammatory bowel disease. Nat Rev Immunol. 2003;3(7):521‐533. 12876555 10.1038/nri1132

[62] Gao F , Chiu S , Motan D , et al. Mesenchymal stem cells and immunomodulation: current status and future prospects. Cell Death Dis. 2016;7(1):e2062‐e2062. 26794657 10.1038/cddis.2015.327 PMC4816164

[63] Li X , Guan Y , Li C , et al. Immunomodulatory effects of mesenchymal stem cells in peripheral nerve injury. Stem Cell Research & Therapy. 2022;13(1):1‐13. 35033187 10.1186/s13287-021-02690-2 PMC8760713

[64] Lim J‐Y , Im K‐I , Lee E‐S , et al. Enhanced immunoregulation of mesenchymal stem cells by IL‐10‐producing type 1 regulatory T cells in collagen‐induced arthritis. Sci Rep. 2016;6(1):1‐13. 27246365 10.1038/srep26851 PMC4887998

[65] Weiss ARR , Dahlke MH . Immunomodulation by mesenchymal stem cells (MSCs): mechanisms of action of living, apoptotic, and dead MSCs. Front Immunol. 2019;10:1191. 31214172 10.3389/fimmu.2019.01191 PMC6557979

[66] Chen ML , Sundrud MS . Cytokine networks and T‐cell subsets in inflammatory bowel diseases. Inflamm Bowel Dis. 2016;22(5):1157‐1167. 26863267 10.1097/MIB.0000000000000714 PMC4838490

[67] Jostins L , Ripke S , Weersma RK , et al. Host‐microbe interactions have shaped the genetic architecture of inflammatory bowel disease. Nature. 2012;491(7422):119‐124. 23128233 10.1038/nature11582 PMC3491803

[68] Strober W , Fuss IJ . Proinflammatory cytokines in the pathogenesis of inflammatory bowel diseases. Gastroenterology. 2011;140(6):1756‐1767. 21530742 10.1053/j.gastro.2011.02.016 PMC3773507

[69] Fuss IJ , Neurath M , Boirivant M , et al. Disparate CD4+ lamina propria (LP) lymphokine secretion profiles in inflammatory bowel disease. Crohn's disease LP cells manifest increased secretion of IFN‐gamma, whereas ulcerative colitis LP cells manifest increased secretion of IL‐5. J Immunol. 1996;157(3):1261‐1270. 8757634

[70] Phinney DG , Pittenger MF . Concise review: MSC‐derived exosomes for cell‐free therapy. Stem Cells. 2017;35(4):851‐858. 28294454 10.1002/stem.2575

[71] Rackov G , Garcia‐Romero N , Esteban‐Rubio S , Carrion‐Navarro J , Belda‐Iniesta C , Ayuso‐Sacido A . Vesicle‐mediated control of cell function: the role of extracellular matrix and microenvironment. Front Physiol. 2018;9:651. 29922170 10.3389/fphys.2018.00651 PMC5996101

[72] Ren S , Chen J , Duscher D , et al. Microvesicles from human adipose stem cells promote wound healing by optimizing cellular functions via AKT and ERK signaling pathways. Stem Cell Res Ther. 2019;10(1):47. 30704535 10.1186/s13287-019-1152-x PMC6357421

[73] Spees JL , Lee RH , Gregory CA . Mechanisms of mesenchymal stem/stromal cell function. Stem Cell Res Ther. 2016;7(1):125. 27581859 10.1186/s13287-016-0363-7 PMC5007684

[74] Ju GQ , Cheng J , Zhong L , et al. Microvesicles derived from human umbilical cord mesenchymal stem cells facilitate tubular epithelial cell dedifferentiation and growth via hepatocyte growth factor induction. PLoS One. 2015;10(3):e0121534. 25793303 10.1371/journal.pone.0121534 PMC4368636

[75] Jansen F , Yang X , Hoelscher M , et al. Endothelial microparticle‐mediated transfer of MicroRNA‐126 promotes vascular endothelial cell repair via SPRED1 and is abrogated in glucose‐damaged endothelial microparticles. Circulation. 2013;128(18):2026‐2038. 24014835 10.1161/CIRCULATIONAHA.113.001720

[76] Lee C , Mitsialis SA , Aslam M , et al. Exosomes mediate the cytoprotective action of mesenchymal stromal cells on hypoxia‐induced pulmonary hypertension. Circulation. 2012;126(22):2601‐2611. 23114789 10.1161/CIRCULATIONAHA.112.114173 PMC3979353

[77] Pan S , Yang X , Jia Y , Li R , Zhao R . Microvesicle‐shuttled miR‐130b reduces fat deposition in recipient primary cultured porcine adipocytes by inhibiting PPAR‐g expression. J Cell Physiol. 2014;229(5):631‐639. 24311275 10.1002/jcp.24486

[78] Legaki E , Roubelakis MG , Theodoropoulos GE , et al. Therapeutic potential of secreted molecules derived from human amniotic fluid mesenchymal stem/stroma cells in a mice model of colitis. Stem Cell Rev Rep. 2016;12(5):604‐612. 27503204 10.1007/s12015-016-9677-1

[79] Fernandez‐Messina L , Gutierrez‐Vazquez C , Rivas‐Garcia E , Sanchez‐Madrid F , de la Fuente H . Immunomodulatory role of microRNAs transferred by extracellular vesicles. Biol Cell. 2015;107(3):61‐77. 25564937 10.1111/boc.201400081 PMC5010100

[80] Ingato D , Lee JU , Sim SJ , Kwon YJ . Good things come in small packages: overcoming challenges to harness extracellular vesicles for therapeutic delivery. J Control Release. 2016;241:174‐185. 27667180 10.1016/j.jconrel.2016.09.016

[81] Luan X , Sansanaphongpricha K , Myers I , Chen H , Yuan H , Sun D . Engineering exosomes as refined biological nanoplatforms for drug delivery. Acta Pharmacol Sin. 2017;38(6):754‐763. 28392567 10.1038/aps.2017.12 PMC5520184

[82] Song Y , Dou H , Li X , et al. Exosomal miR‐146a contributes to the enhanced therapeutic efficacy of interleukin‐1beta‐primed mesenchymal stem cells against sepsis. Stem Cells. 2017;35(5):1208‐1221. 28090688 10.1002/stem.2564

