# Supplementary figures and images for: Effect of Crohn's disease mesenteric mesenchymal stem cells and their extracellular vesicles on T‐cell immunosuppressive capacity
Source: J Cell Mol Med. 2022 Sep 1;26(19):4924–39. doi: 10.1111/jcmm.17483 (PMC9549497; doi:10.1111/jcmm.17483)

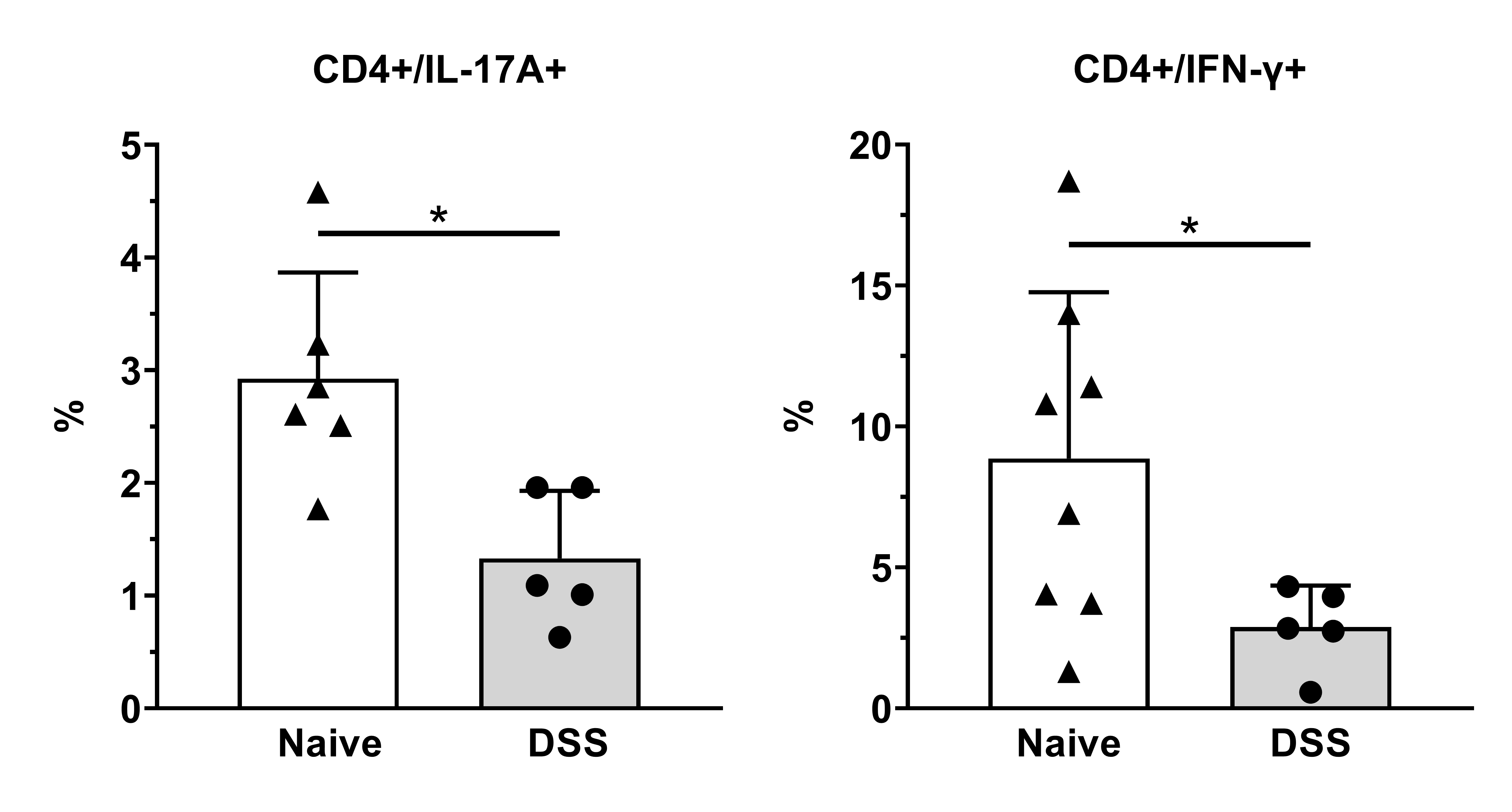

Supplement: Supplementary file 1 — Figure S1 [file JCMM-26-4924-s003.tif]

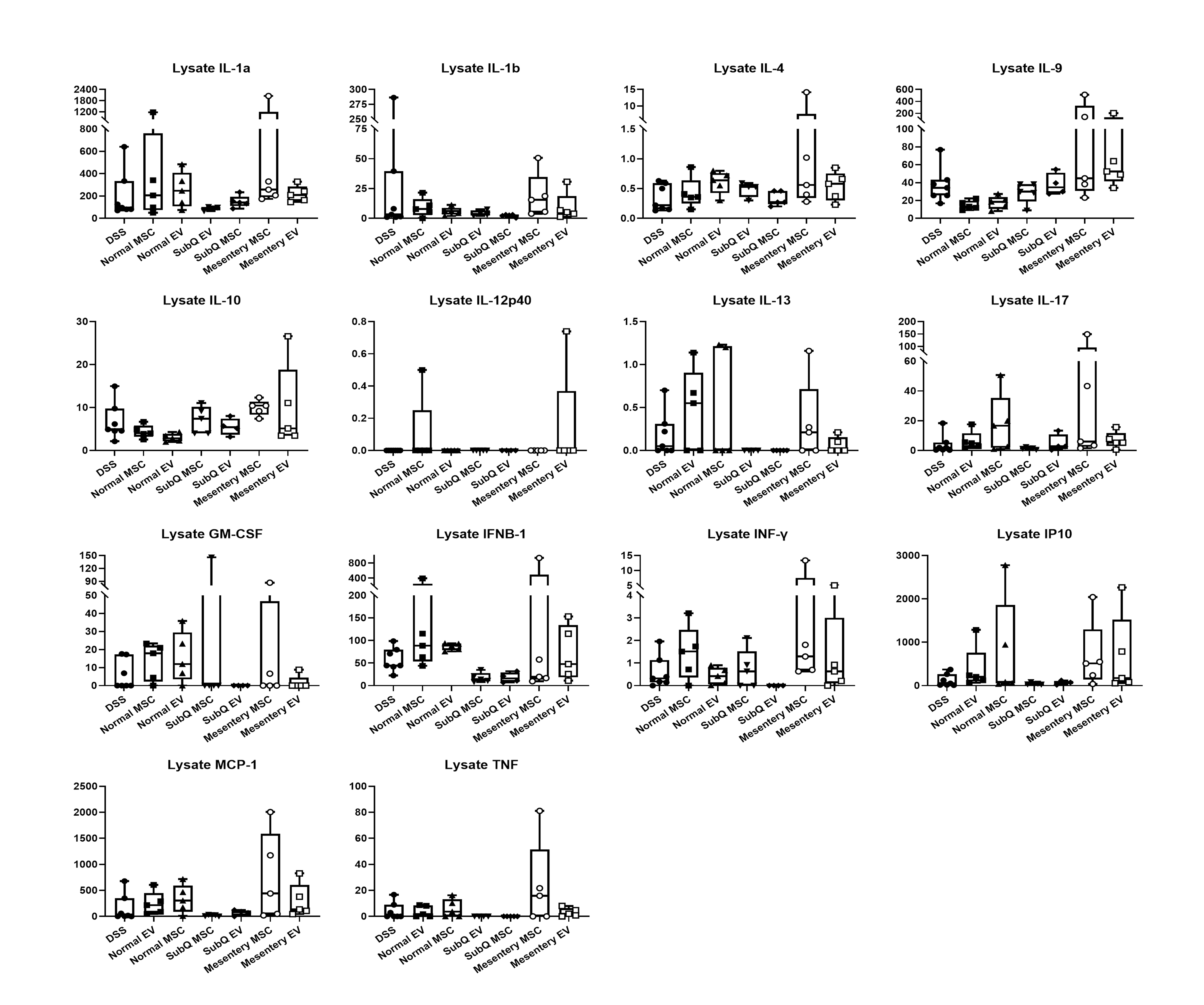

Supplement: Supplementary file 2 — Figure S2 [file JCMM-26-4924-s002.tif]
