# Supplementary material for: Effect of Crohn's disease mesenteric mesenchymal stem cells and their extracellular vesicles on T‐cell immunosuppressive capacity
Source: J Cell Mol Med. 2022 Sep 1;26(19):4924–39. doi: 10.1111/jcmm.17483 (PMC9549497; doi:10.1111/jcmm.17483)
Supplement: Supplementary file 3 — Table S1–S2 [file JCMM-26-4924-s001.docx]

***Table 1. Patient Characteristics***

| ***Patient*** | ***Diagnosis*** | ***Age*** | ***Sex*** | ***BMI*** | ***Phenotype of CD*** | ***Corticosteroids at surgery*** | ***Monoclonal antibody at surgery*** |
| --- | --- | --- | --- | --- | --- | --- | --- |
| Patient 1 | Crohn’s disease | 62 | F | 25.3 | Stricturing | Yes | Yes, Certolizumab |
| Patient 2 | Crohn’s disease | 50 | F | 50.8 | Inflammatory | No | No |
| Patient 3 | Crohn’s disease | 29 | F | 24.6 | Inflammatory | No | Yes, Ustekinumab |
| Patient 4 | Crohn’s disease | 42 | M | 25.9 | Stricturing | No | No |
| Patient 5 | Crohn’s disease | 26 | F | 23.1 | Stricturing | No | Yes, Ustekinumab |
| Patient 6 | Crohn’s disease | 36 | M | 20 | Stricturing | No | Yes, Ustekinumab |
| Patient 7 | Crohn’s disease | 40 | F | 20.4 | Inflammatory | Yes | No |
| Patient 8 | Crohn’s disease | 66 | F | 28 | Inflammatory | Yes | No |
| Patient 9 | Control | 51 | M | 32.9 | N/A | No | No |
| Patient 10 | Control | 33 | M | 23.1 | N/A | No | No |
| Patient 11 | Control | 49 | F | 47.1 | N/A | No | No |
| Patient 12 | Control | 62 | F | 25.4 | N/A | No | No |

Table 2. Disease Activity Index Scoring Chart

| ***Clinical score*** | ***Weight loss (%)*** | ***Stool consistency*** | ***Hematochezia*** |
| --- | --- | --- | --- |
| 0 | None | Normal | None |
| 1 | 1–10% | Soft stool | Hemaoccult positive |
| 2 | 10–20% | Diarrhea | Gross blood |
| 3 | 20% | Diarrhea | n/a |

Table 3. Disease activity index score for DSS-induced colitis mice treated with normal, SubQ or Mes MSCs or EVs.

| ***Group*** | ***D0*** | ***D5*** | ***D6*** | ***D7*** | ***D9*** | ***D10*** |
| --- | --- | --- | --- | --- | --- | --- |
| PBS | 0 | 0.75±0.26 | 1.31±0.52 | 2.65±0.15 | 2.88±0.37 | 2.99±0.14 |
| SubQ MSC | 0 | 0.18±0.09 ^a^ | 0.49±0.17 ^a^ | 0.75±0.3 ^a^ | 1.11±0.25 ^a^ | 1.64±0.37 ^a^ |
| SubQ EV | 0 | 0.21±0.08 ^a^ | 0.54±0.13 ^a^ | 0.71±0.28 ^a^ | 1.22±0.24 ^a^ | 1.88±0.65 ^a^ |
| Mes MSC | 0 | 0.71±0.13 ^a^ | 0.98±0.67 ^a^ | 1.96±0.95 ^a^ | 2.78±0.46 ^a^ | 2.95±0.31 ^a^ |
| Mes EV | 0 | 0.59±0.25 ^a^ | 1.22±0.15 ^a^ | 2.21±0.56 ^a^ | 2.92±0.35 ^a^ | 2.97±0.12 ^a^ |
| Normal MSC | 0 | 0.19±0.12 ^a^ | 0.52±0.27 ^a^ | 0.66±0.42 ^a^ | 1.32±0.41 ^a^ | 1.96±0.41 ^a^ |
| Normal EV | 0 | 0.28±0.11 ^a^ | 0.69±0.22 ^a^ | 0.98±0.22 ^a^ | 1.62±0.69 ^a^ | 2.12±0.72 ^a^ |

*D: day; SubQ: subcutaneous; MSCs: Mesenchymal stem cells, EVs: Extracellular Vesicle; MES: Mesentery. Values are presented as mean only or mean ± SEM.* *^a^ p<0.05.*

***Supplement Table 1.*** Activation z-score, p-value, and number of differentially expressed genes present in Crohn’s subcutaneous versus Crohn’s mesentery analysis of selected Diseases and Functions and Canonical Pathways.

| **Diseases and Functions** | Activation z-score | p-value | Number of DEG |
| --- | --- | --- | --- |
| T cell response | 2.264 | 3.12E-22 | 56 |
| Infiltration by T lymphocytes | 2.978 | 2.59E-20 | 51 |
| T cell development | 6.505 | 6.03E-53 | 175 |
| T cell homeostasis | 6.544 | 5.48E-55 | 180 |
| T cell migration | 6.039 | 2.54E-37 | 104 |
| Interaction of T lymphocytes | 5.687 | 3.05E-28 | 60 |
| Differentiation of T lymphocytes | 5.641 | 6.28E-42 | 130 |
| Quantity of regulatory T lymphocytes | 3.702 | 1.49E-20 | 49 |
| Quantity of T lymphocytes | 4.418 | 5.9E-61 | 192 |
| Activation of T lymphocytes | 4.222 | 3.22E-54 | 145 |
| Stimulation of T lymphocytes | 4.03 | 8.33E-21 | 46 |
| Adhesion of T lymphocytes | 4.784 | 5.2E-20 | 40 |
| Cell proliferation of T lymphocytes | 4.721 | 4.23E-70 | 213 |
| Cell movement of T lymphocytes | 5.295 | 3.41E-32 | 91 |
| Binding of T lymphocytes | 5.179 | 2.44E-28 | 57 |
| **Canonical Pathways** |  |  |  |
| T Cell Receptor Signaling | 6.364 | 7.14E-9 | 74 |
| Regulation of IL-2 Expression in Activated and Anergic T Lymphocytes | 5.516 | 2.86E-2 | 38 |
| PKCθ Signaling in T Lymphocytes | 4.747 | 5.75E-5 | 57 |
| Systemic Lupus Erythematosus In T Cell Signaling Pathway | 3.888 | 2.15E-4 | 61 |
| Calcium-Induced T Lymohocyte Apoptosis | 4.146 | 2.47E-4 | 47 |
| NUR77 Signaling in T Lymphocytes | 3.051 | 6.42E-3 | 45 |

***Supplement Table 2. T cell Polarization Stimuli Combination***

|  | rmIL-2 | rmIL-12 | rmIL-6 | rmTGF-β | anti-mIFN-γ | anti-mIL-4 |
| --- | --- | --- | --- | --- | --- | --- |
| Th1 | 20 ng/mL | 25 ng/mL | N/A | N/A | N/A | 10 μg/mL |
| Th2 | N/A | 10 μg/mL | N/A | N/A | 10 μg/mL | 10 μg/mL |
| Th9 | N/A | N/A | N/A | 3 ng/mL | N/A | 10 ng/mL |
| Th17 | N/A | N/A | 20 ng/mL | 5 ng/mL | 10 μg/mL | 10 μg/mL |
| Treg | 10 ng/mL | N/A | N/A | 0.2 ng/ml | N/A | N/A |

r: recombinant, m: mouse
